# *Botrytis cinerea* Protein *O*-Mannosyltransferases Play Critical Roles in Morphogenesis, Growth, and Virulence

**DOI:** 10.1371/journal.pone.0065924

**Published:** 2013-06-06

**Authors:** Mario González, Nélida Brito, Marcos Frías, Celedonio González

**Affiliations:** Departamento de Bioquímica y Biología Molecular, Universidad de La Laguna, La Laguna (Tenerife), Spain; University of Wisconsin - Madison, United States of America

## Abstract

Protein *O*-glycosylation is crucial in determining the structure and function of numerous secreted and membrane-bound proteins. In fungi, this process begins with the addition of a mannose residue by protein *O*-mannosyltransferases (PMTs) in the lumen side of the ER membrane. We have generated mutants of the three *Botrytis cinerea pmt* genes to study their role in the virulence of this wide-range plant pathogen. *B. cinerea* PMTs, especially PMT2, are critical for the stability of the cell wall and are necessary for sporulation and for the generation of the extracellular matrix. PMTs are also individually required for full virulence in a variety of hosts, with a special role in the penetration of intact plant leaves. The most significant case is that of grapevine leaves, whose penetration requires the three functional PMTs. Furthermore, PMT2 also contributes significantly to fungal adherence on grapevine and tobacco leaves. Analysis of extracellular and membrane proteins showed significant changes in the pattern of protein secretion and glycosylation by the *pmt* mutants, and allowed the identification of new protein substrates putatively glycosylated by specific PMTs. Since plants do no possess these enzymes, PMTs constitute a promising target in the development of novel control strategies against *B. cinerea*.

## Introduction

Protein glycosylation is an important and highly conserved posttranslational modification consisting in the addition of carbohydrate residues to target proteins. It plays essential roles in eukaryotic cells from fungi to mammals [1], being crucial in determining the structure and function of numerous secreted and membrane-bound proteins. Glycosyl residues, mainly *N*-acetylglucosamine, *N*-acetylgalactosamine, mannose, galactose or glucose, can be linked to proteins via asparagine (*N*-glycosylation) or via hydroxylated amino acids including serine, threonine, and, more rarely, tyrosine, hydroxyproline and hydroxylysine (*O*-glycosylation). Both glycosylation pathways are conserved from prokaryotes to humans, but some differences exist in the class and number of the carbohydrate residues added [2], [3]. *N*-linked oligosaccharides are diverse in the type of sugars added but, in eukaryotes, they are all bound by a GlcNAc monomer to a defined sequence in proteins, Asn-X-Ser/Thr [3], where X can be any amino acid but Pro. In contrast, *O*-linked carbohydrate chains are more heterogeneous in relation to their sugar components and the way they bind to one another and to the proteins [1]–[3]. Currently there is no known consensus sequence for protein *O*-glycosylation sites, although prediction tools have been developed for mammals [4] and it is generally accepted that the presence of regions rich in Ser/Thr residues in secretory proteins results in the addition of a high density of *O*-linked oligosaccharides [5]–[7].

*O*-glycosylation has been studied extensively in fungi, mainly in the budding yeast *Saccharomyces cerevisiae* [1], [8]–[10]. The first step of the *O*-glycosylation pathway in these organisms, often referred to as *O*-mannosylation, consists in the addition of one mannose residue to Ser/Thr residues in target proteins catalyzed by dolichyl phosphate mannose-dependent protein *O*-mannosyltransferases (PMTs), located in the lumen side of the endoplasmic reticulum (ER) membrane [11]. However, the initial addition of glucose or galactose residues to Ser/Thr has also been reported for *Trichoderma* [3]. The oligosaccharide chain is then extended, as the protein continues the secretion through Golgi, by several other enzymes generating linear or branched sugar chains composed mostly of mannose residues [3], [9]. Yeast usually have linear sugar chains composed exclusively of up to five mannose residues, but filamentous fungi may have branched chains containing also glucose or galactose [3], [11]. PMTs have been found in both prokaryotes and eukaryotes [11], but not in plants, making these proteins promising targets in the design of novel control strategies against fungal plant-pathogens.

Phylogenetic analysis has shown that PMTs can be classified into three subfamilies: PMT1, PMT2 and PMT4 [12], [13]. *S. cerevisiae* and *Candida albicans* genomes both code for one member of subfamily 4 and 2–3 members of subfamilies 1 and 2, but *Schizosaccharomyces pombe* and all filamentous fungi analyzed so far have only one representative *pmt* gene per subfamily [1]. All PMTs are ER integral membrane proteins and share similar hydropathy profiles, seven transmembrane domains have been proposed [14], as well as the presence of three MIR conserved motifs essential for the mannosyltransferase activity [12]. In *S. cerevisiae*, proteins of the PMT1 subfamily (Pmt1p and Pmt5p) form heterodimers with proteins belonging to the PMT2 subfamily (Pmt2p and Pmt3p), whereas PMT4 acts as homodimer [15]. Although bioinformatics approaches have been used to identify putative targets of specific PMTs [5], [6], only a few proteins have been identified as substrates of specific PMTs in yeast [5] and in filamentous fungi [6], [16].

The physiological role of *O*-glycosylation in fungi has been established primarily through the study of *pmt* mutants, since they are blocked in the first step of *O*- mannosylation. As a result, it is known that *O*-glycosylation is crucial for these organisms, as no mutant defective in all *pmt* genes could be isolated so far. Moreover, the deletion of single *pmt* genes, usually the one coding for a PMT2 subfamily protein, or the simultaneous deletion of several of them, can also result in lack of viability. Those single or multiple *pmt* mutants which are viable often display clear phenotypes whose study has allowed the establishment of important roles for *O*-glycosylation in cell wall integrity, cell morphology and proper development, sensing environmental stress, as well as in the stability, sorting, and localization of secretory proteins [10], [11], [17]–[20]. In *S. cerevisiae*, single *pmt* mutants are viable but the simultaneous disruption of three *pmt* genes, one member of each subfamily, is lethal [8]. In yeast with a single *pmt* gene per subfamily such as C*ryptococcus neoformans* [21], the subfamily 2 gene is essential for growth, although contradictory results have been reported for *S. pombe* [22], [23]. Single *S. pombe* mutants in the genes of subfamilies 1 or 4, although viable, show a pleiotropic phenotype with altered cell morphology, abnormal cell wall composition, and defective cell-cell separation and mating [22], [23]. In the case of the human pathogen *C. neoformans* deletion of subfamily 1 or 4 genes also resulted in altered stress resistance and reduced virulence [21]. *S. pombe* and *C. neoformans* mutants lacking both genes from subfamilies 1 and 4 are also not viable [21], [22]. The situation is similar in filamentous fungi where the PMT2 subfamily member is either essential, as in *Ustilago maydis* [24], or its deletion has a great impact in viability, as in *Aspergillus nidulans* [25]. Interestingly, deletion of the gene for the single PMT4 in *U. maydis* completely abolished pathogenicity of the fungus by eliminating the ability to penetrate the plant tissue, without otherwise affecting the *U. maydis* life cycle [24].

We have previously noted the abundance of Ser/Thr rich regions among *Botrytis cinerea* secreted proteins [7], [26], which were predicted to be the site of high density *O*-glycosylation, pointing to this process as an important determinant in the biology and virulence of this widely distributed and crop-devastating plant pathogen. Here we report the characterization of the three *B. cinerea pmt* genes and their contribution to hyphae morphology, growth, and virulence, as well as the identification of putative specific substrates of individual PMTs.

## Results

### The Three *B. cinera pmt* Genes are Constitutively Expressed

**Figure 1:**
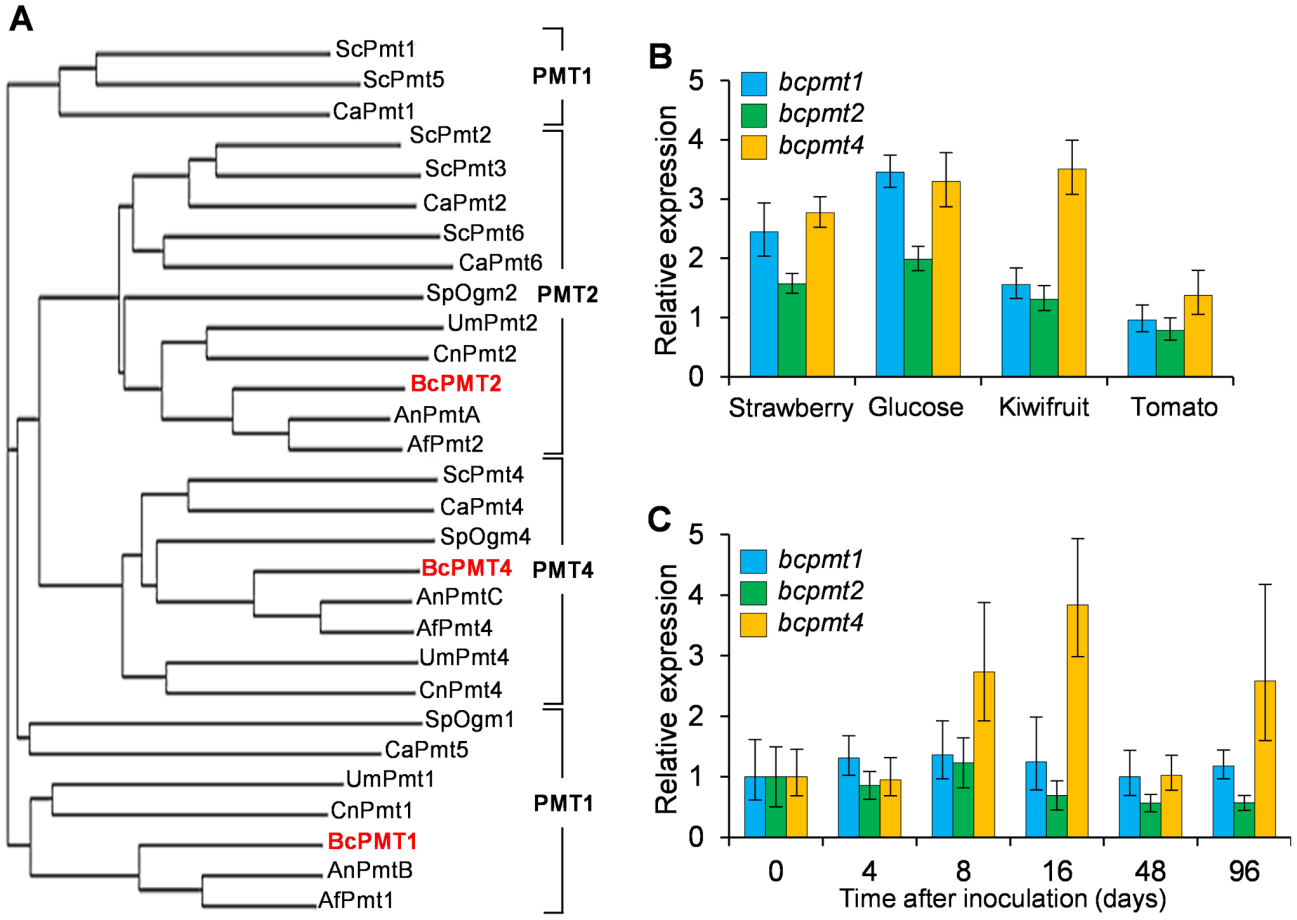
Phylogeny and expression of *B. cinerea* PMTs. **A**) Phylogenetic analysis comparing *B. cinerea* PMTs with those of various fungi. First two letters in each protein name indicates the fungus it comes from (Sc: *Saccharomyces cerevisiae*; Ca: *Candida albicans*; Sp: *Schizosaccharomyces pombe*; Um: *Ustilago maydis*; Cn: *Cryptococcus neoformans*; Bc: *Botrytis cinerea*; An: *Aspergillus nidulans*; Af: *Aspergillus fumigatus*). Accession numbers can be found in Table S1. B) and C) Q-RT-PCR analysis of *bcpmt* expression in the wild-type strain B05.10 grown either in liquid media supplemented with the indicated plant extracts or glucose (B), or in *B. cinerea*-infected tomato leaves (C). Bars represent the relative amount of mRNA with respect to ungerminated conidia as mean values (n = 3) and standard deviation.

A BLAST search of the *B. cinerea* T4 (http://urgi.versailles.inra.fr/Species/Botrytis) and B05.10 (http://www.broadinstitute.org/annotation/genome/botrytis_cinerea) genome databases, using *S. cerevisiae* PMT proteins as query sequences, allowed the identification of three putative PMT homologues. Phylogenetic analysis with CLUSTALW2 (Figure 1A) grouped each of the three putative *B. cinerea* PMTs into each PMT subfamily [1], and we therefore named these genes as *bcpmt1* (BofuT4_P160540.1/BC1G_12913.1, subfamily PMT1), *bcpmt2* (BofuT4_P003410.1/BC1G_02981.1, subfamily PMT2) and *bcpmt4* (BofuT4_P109250.1/BC1G_02548.1, subfamily PMT4). As the rest of the filamentous fungi studied so far, *B. cinerea* has a single representative of each PMT subfamily.

The comparison of the hydropathy profiles of each BcPMT with a consensus hydropathy profile for the corresponding PMT subfamily (Figure S1) revealed that *B. cinerea* PMTs are structurally similar to the rest of fungal PMTs, and are probably inserted in a similar way in the ER membrane. Although *in silico* prediction tools (TMHMM [27], PredictProtein [28], [29], or Phobius [30]) predicted up to 12 transmembrane domains in the 3 BcPMTs (Figure S1), the coincidence of the hydropathy profiles with those of *S. cerevisiae* PMTs, which have been reported to have 7 transmembrane domains [14], and the well-known fact that protein structure is much more conserved throughout evolution than amino acid sequence [31], led us to hypothesize the same distribution of transmembrane domains (Figure S1).

The PMT and MIR domains typical of PMTs [11] are also present in the *B. cinerea* enzymes (Figure S1), and there is strong sequence similarity in the so-called loops 1 and 5 (Figure S2) with a percentage of identity always higher than 50% in a comparison with the corresponding loops of *S. cerevisiae* PMTs (not shown). These loops are located in the ER lumen and have been implicated in the mannosyl transferase activity [11]. The essential DE motif in loop 1 suggested to play a central role in catalysis [32] is also present in the three BcPMTs, as all the conserved residues and sequence motifs identified as essential for catalysis or dimerization by Girrbach *et al*. [12].

Expression analysis of the three *bcpmt* genes reveled that all of them were expressed under all conditions tested (Figure 1B). In minimal media supplemented with various plant extracts, or in the control media with glucose alone, the levels of expression were not very different from one another, ranging between 1 and 4 times the amount of mRNA present in ungerminated conidia. All 3 genes were also expressed *in planta*, in tomato leaves infected with conidia of *B. cinerea* B05.10 (Figure 1C), and the time course of the expression indicated a relatively steady pattern of expression for all three genes, with only a small increase in *bcpmt4* expression at 8 and 16 hours post-inoculation.

### All *bcpmt* Mutant Strains Show Reduced Growth in Axenic Culture

Single knock-out mutants were generated for all three *bcpmt* genes by replacing the coding sequence with a hygromycin resistance cassette (Figure S3) and homokaryotic strains were identified by PCR and Southern-blot analysis (Figure S3). The resulting single *bcpmt* mutant strains were designated Δ*bcpmt1*, Δ*bcpmt2*, and Δ*bcpmt4*.

**Figure 2:**
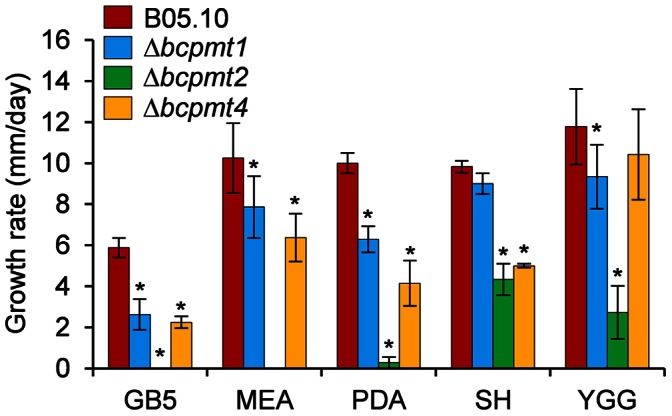
Growth of Δ*bcpmt* strains in axenic culture. Growth rates (mean values and standard deviation) of the three mutant strains and the wild type (B05.10) in different media. Media compositions are given in Experimental Procedures. Asterisks indicate a statistically significant difference with the wild type in the same medium (n≥3, p<0.05).

**Figure 3:**
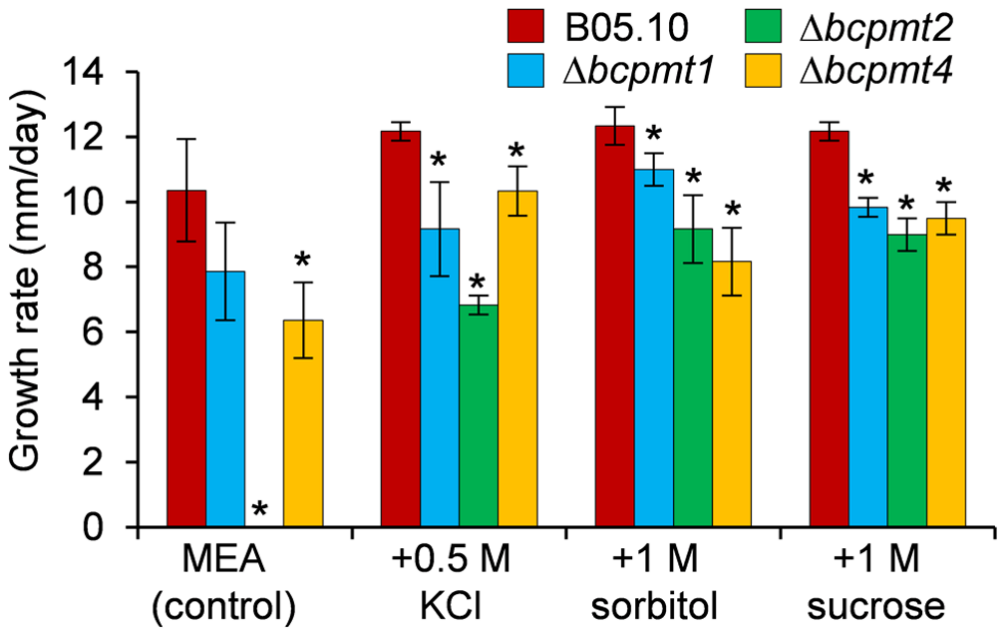
Effect of osmotic stabilizers on the growth of Δ*bcpmt* mutants. Growth rates (mean values and standard deviation) obtained in MEA plates supplemented with the indicated osmotic agents. Asterisks indicate a statistically significant difference with the wild type in the same medium (n = 3, p<0.05).

All three Δ*bcpmt* mutants showed a lower growth rate than the wild-type strain in all media tested (Figure 2). Δ*bcpmt2* was not able to grow at all in 3 of the most standard *B. cinerea* growth media, Gamborg’s B5, MEA and PDA, while Δ*bcpmt1* and Δ*bcpmt4* grew in these three media with a 23–62% reduction in the growth rate, as compared with the wild-type strain. These differences were smaller in the richer medium YGG and in SH, and these media even allowed the growth of Δ*bcpmt2*. Other studies in yeast and filamentous fungi have shown that *pmt* mutants partially recover the normal growth rate levels in media supplemented with osmotic stabilizers [8], [33], [34]. The addition of 1 M sorbitol, 1 M sucrose, or 0.5 M KCl to the cultures in MEA (Figure 3) increased the growth rate of the wild-type strain B05.10, surprisingly, by 18%. This increase was only slightly higher for Δ*bcpmt1* (17–40%), but a clearer recovery of the growth rate was observed in the case of Δ*bcpmt4* (28–62%), and especially in the case of Δ*bcpmt2* (Figure 3), which did not growth at all in MEA but recovered the growth rate to 56–74% of that of the wild type with the osmotic stabilizers. The stabilizing effect of the increased osmolarity is usually interpreted by a weakened cell wall structure. A cell wall not fully able to cope with the osmotic pressure would be a growth-limiting factor at low osmolarities, but not so when osmolarities of the cell interior and the surrounding medium are similar. The fact that it is especially Δ*bcpmt2* the mutant that most clearly recovers the growth rate in the presence of osmotic stabilizers may represent that BcPMT2 is the main contributor to the *O*-glycosylation of key structural cell-wall proteins.

### Δ*bcpmt* Strains Produce Less Conidia but More Sclerotia

**Figure 4:**
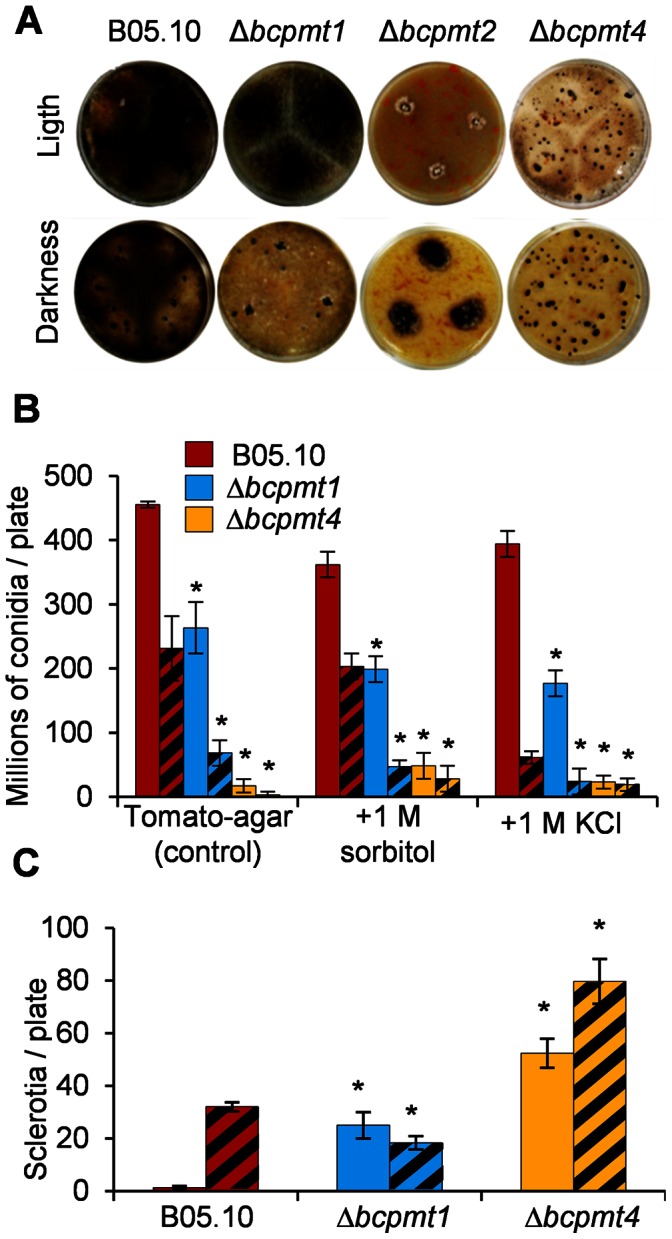
Conidia and sclerotia production by Δ*bcpmt* mutants. The indicated strains were grown under continuous light (plain bars) or in darkness (hatched bars) in tomato-agar plates. Osmotic agents were added when indicated. **A**) Appearance of the colonies 12 days after inoculation. **B**) Conidia production at day 15. **C**) Sclerotia production at day 15. Bars represent mean values (n = 3) and standard deviation, and asterisks indicate a statistically significant difference with the wild type in the same medium (p<0.05).

The amount of conidia and sclerotia produced was determined quantitatively for the Δ*bcpmt* mutants grown for 15 days on tomato fruit extract, either under continuous visible light, to maximize the production of conidia, or in the dark, to maximize the production of sclerotia [35]. Δ*bcpmt2* was completely unable to sporulate, even when osmotic stabilizers were added to the growth medium, and no clear sclerotia formation was observed (Figure 4). However, Δ*bcpmt2* colonies were strongly melanized in the dark, which is one of the features of sclerotia (Figure 4A). Δ*bcpmt4* and especially Δ*bcpmt1* were able to sporulate, but the number of conidia produced was reduced (Figure 4B). This difference was larger in the dark, so that light was a more potent inducer of sporulation for the *pmt* mutants than for the wild type. Addition of sorbitol improved sporulation only slightly in the case of Δ*bcpmt4* (Figure 4B). Conidia obtained from these two mutants did not show any difference in their germination rate as compared with the wild type (not shown), about 83% of conidia obtained were able to germinate in all cases. Sclerotia production, on the contrary, was higher for Δ*bcpmt1* and Δ*bcpmt4*, under continuous illumination, than for the wild type (Figure 4C). Light is a strong repressor of sclerotia formation for *B. cinerea* [35] but it seems that it does not produce the same effect in the *pmt* mutants. Addition of the osmotic stabilizers KCl and sorbitol completely abolished sclerotia formation for the three mutants and for the wild type (not shown). The sclerotia produced were viable in 100% of the cases.

### Δ*bcpmt2* Shows Sensibility to Calcofluor White, SDS and Culture Shaking

**Figure 5:**
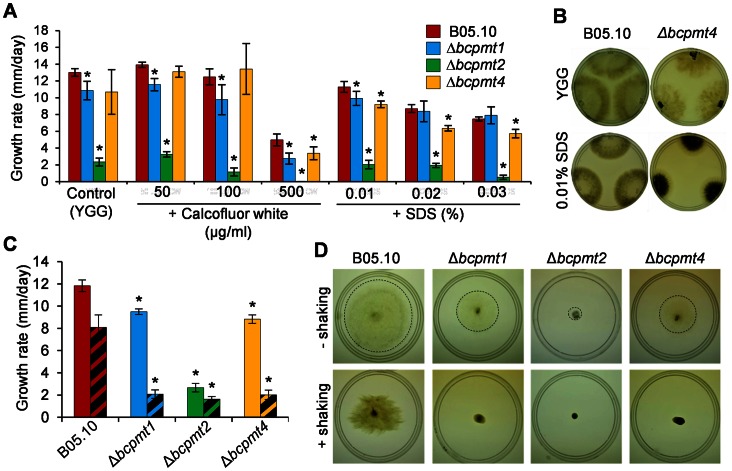
Effect of various chemical agents and culture shaking on the growth of Δ*bcpmt* mutants. **A** **B**) Dark pigmentation of Δ*bcpmt4* in YGG-agar supplemented with 0.01% SDS. **C**) and **D**) Effect of culture shaking on growth rate (C) and colony morphology (D). The indicated strains were grown in liquid YGG with (hatched bars), or without (plain bars), shaking (60 rpm in a reciprocal shaker). Pictures were taken at day 4. A dashed line was drawn at the edge of colonies, where necessary, to aid visualization. Bars represent mean values (n = 6) and standard deviation, and asterisks indicate a statistically significant difference with the wild type in the same conditions (p<0.05).

To test the integrity of the cell wall, growth rates were measured for the Δ*bcpmt* mutants in YGG plates, a medium that allowed the growth of the three mutants, supplemented with different concentrations of calcofluor white (CW), Congo red (CR), or sodium dodecyl sulphate (SDS). 500 µg ml^−1^ CW inhibited the growth of the wild-type strain and the three mutants (Figure 5A), but the inhibitory effect was stronger for Δ*bcpmt1,* Δ*bcpmt4*, and especially for Δ*bcpmt2*, which was also affected at lower concentrations. Similarly, addition of SDS resulted in significant growth inhibition only for Δ*bcpmt2* (Figure 5A). At 0.03% SDS, the growth rate of Δ*bcpmt2* is reduced 77% as compared to the growth without SDS, while the reduction observed for the wild-type strain was 42%. In addition, Δ*bcpmt4* showed a change in its pigmentation induced by SDS (Figure 5B). Interplay between the cell wall integrity pathway and melanin biosynthesis genes has been proposed for *B. cinerea* [36], a fact that may be related with this increased pigmentation. Contrary to what has been reported for some fungi [25], [37], but in accordance to others [24], the addition of CR to YGG plates did not cause any reduction in the growth rates of the wild-type strain or any of the Δ*bcpmt* mutants (not shown). It is necessary to note, however, that those *pmt* mutants with increased sensitivity to CR, in other fungi, show it usually at temperatures higher than what is optimal for the corresponding organism [25].

A weakened cell wall may also represent a problem for fungi growing under some kind of mechanical stress. This was tested simply by growing the Δ*bcpmt* mutants in liquid YGG medium, with or without shaking, and comparing the size of the colonies formed by the mycelium expanding from individual agar plugs. The slow shaking used (60 rpm in a reciprocal shaker) caused a reduction in the colony size for every strain, included the wild type (Figure 5C and D), possibly because the turbulence in the medium makes the whole colony to be more like a wool yarn instead of the typical star-like morphology found on agar plates. This reduction, however, was much more pronounced for Δ*bcpmt1* and Δ*bcpmt4* than for the wild type, suggesting that the mechanical resistance, and therefore the structural integrity of the cell wall and/or extracellular matrix, is affected in these two mutants. The reduction in colony size caused by agitation was very small for the Δ*bcpmt2* mutant, but it is necessary to note that the colonies of this mutant are also very small and compact on agar and the mycelium shows an aberrant morphology, as will be explained below.

Increased sensitivity to cell wall degrading enzymes can also be taken as an indication of a weakened cell wall, and this was checked for the Δ*bcpmt* mutants by estimating the rate of protoplast generation from mycelium. Incubation with a mixture of enzymes displaying β-glucanase, cellulase, protease, and chitinase activities, according to the manufacturer (Lysing Enzymes from *Trichoderma harzianum*, SIGMA L1412), resulted in the release of protoplast at an increased rate from the three mutants, as compared with the wild type (not shown). Δ*bcpmt1* and Δ*bcpmt2* released about twice the number of protoplast per gram of dry weight released by the wild type, and Δ*bcpmt4* released about three fold.

Taken together, all these results imply that all three BcPMT proteins contribute, one way or another, to the structure, stability, or rigidity of the *B. cinerea* cell envelopes. It has been known for long that *O*-glycosylated proteins play an important role in maintaining the structure of the fungal cell wall [11], [38], and this may also be true for the extracellular matrix [39]. Our results suggest that in *B. cinerea* the glycosylation of this kind of proteins is carried out by the three PMTs.

### Δ*bcpmt* Strains have Atypical Hyphal Morphology and Produce Less Extracellular Matrix

**Figure 6:**
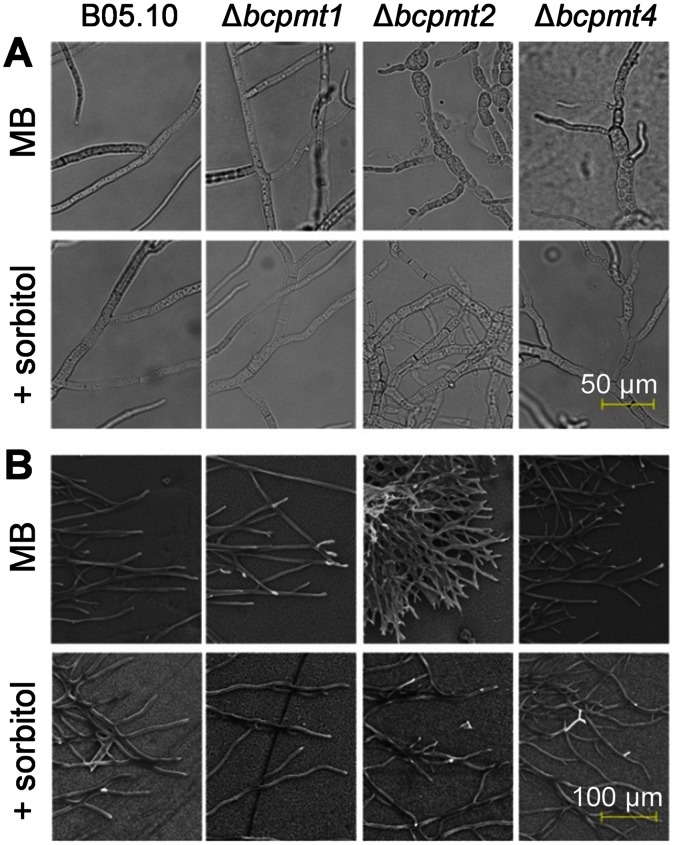
Hyphal morphology of the Δ*bcpmt* mutant strains. The three mutants and the wild type (B05.10) were grown in MB. 1 M sorbitol was added to the medium where indicated. **A**) Morphology under the microscope. **B**) Morphology under the scanning electron microscope.

Microscopic observation of Δ*bcpmt* mutants, grown in the chemically defined medium MB supplemented, or not, with 1 M Sorbitol, revealed big morphological changes in the hyphae of Δ*bcpmt2* and Δ*bcpmt4*, especially the former (Figure 6). The most significant of these changes was the increase in the thickness of cells and the presence of balloon-like swollen cells in these two mutants, which partially disappear when 1 M sorbitol was included in the culture (Figure 6A). As discussed before, this can be explained by a softened cell wall not able to withstand the osmotic pressure. Similar morphologies have also been observed in *pmt* mutants in *Asperglillus awamori* [16], *A. nidulans* [33], [40], *A. fumigatus* [41] or *Neurospora crassa* [42]. Curiously, Δ*bcpmt2* also displayed a more frequent appearance of septa separating individual cells (Figure 6A), especially evident when staining with CW (not shown). The branching pattern was also different for Δ*bcpmt2* and Δ*bcpmt4*, with a higher number of branching points in both of them (Figure 6B). Overall, it seems that Δ*bcpmt1* is morphologically similar to the wild type, Δ*bcpmt2* presents swollen, more frequently septated, and hyperbranched hyphae forming compact colonies, and Δ*bcpmt4* displays morphological characteristics which are intermediate between the wild type and Δ*bcpmt2*. All these changes, with the clear exception of the increase in septum frequency, reverted with the addition of the osmotic stabilizer sorbitol, indicating that they are probably the consequence of the osmotic pressure acting on a weakened cell wall.

**Figure 7:**
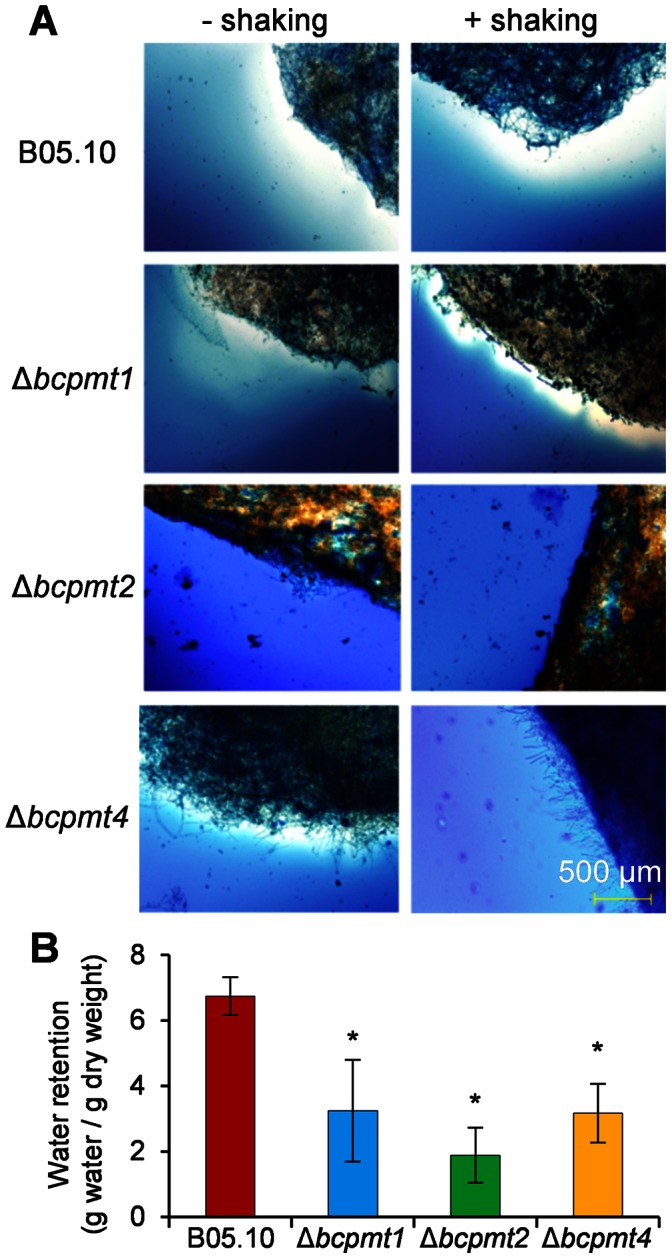
Effect of the mutation of *bcpmt* genes on the production of extracellular matrix. **A**Δ*bcpmt* mutants and the wild type (B05.10) grown in liquid YGG medium with or without shaking (60 rpm in a reciprocal shaker). Extracellular matrix is negatively stained against the India ink background. **B**) Water retention capabilities of the Δ*bcpmt* mutants. All strains were grown in liquid YGG for 4 days without shaking, from a single agar plug, filtered for exactly 2.5 min on pre-weighted filter papers, weighed to determine wet weight, dried to completion at 60°C, and reweighed to determined dry weight. Water retention (difference between dry and wet weight) was normalized to dry weight. Bars represent mean values (n = 3) and standard deviation, and asterisks indicate a statistically significant difference with the wild type (p<0.05).

*B. cinerea* hyphae are surrounded by a very prominent extracellular matrix (ECM) [39], [43], especially in very rich media, which can be visualized as a white halo around fungal colonies stained with India ink [44]. To analyze ECM in the Δ*bcpmt* mutants, fungal strains were grown in the rich medium YGG, stained, and observed under the microscope (Figure 7A). In cultures without shaking, where ECM would be more-easily retained by the mycelium, the matrix seems to be absent in Δ*bcpmt2*, and reduced to some extent in Δ*bcpmt4* as compared to the wild type. When the fungus was grown with slow agitation (60 rpm in a reciprocal shaker) the differences became more obvious and both Δ*bcpmt1* and Δ*bcpmt4,* besides Δ*bcpmt2*, displayed less matrix than the wild type, almost nothing in the case of Δ*bcpmt4*.

One of the proposed functions of extracellular polysaccharides is the retention of water in the vicinity of the cells [45]. A common observation is that mucilaginous old cultures of *B. cinerea* grown in rich media are much more difficult to desiccate on filter paper than young cultures on minimal medium. We took advantage of this observation to indirectly estimate the amount of ECM, by measuring the water retention capacity of the mycelium grown in rich medium. The results (Figure 7B) indicate that Δ*bcpmt2* has a water retention capability quite smaller than the wild type, while Δ*bcpmt1* and Δ*bcpmt4* show and intermediate value, in good agreement with the amount of ECM revealed by India ink staining.

These results point to *O*-glycosylated proteins as key components of ECM, a hypothesis that was further explored by treating ECM-rich mycelium with proteinase K (Figure S4). The protease treatment partially disintegrates ECM in the wild-type strain, and does more so in the Δ*bcpmt4* mutant, thus confirming the importance of proteins as constituents of ECM.

### The Three Δ*bcpmt* Mutants are Less Virulent; Δ*bcpmt2* and Δ*bcpmt4* have a Reduced Ability to Penetrate the Plant Tissue

**Figure 8:**
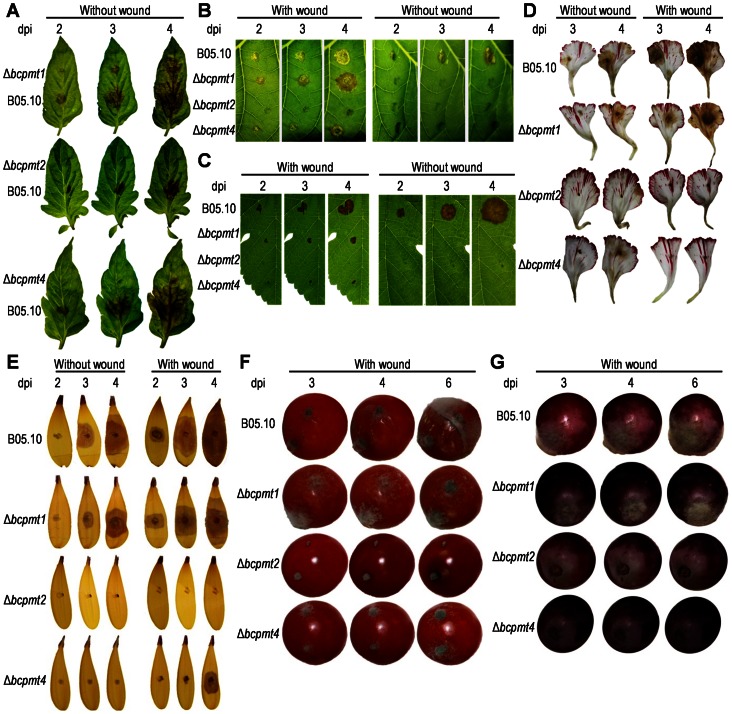
Virulence of Δ*bcpmt* mutants on different plant tissues. Inoculations were done with 0.2 mm agar cubes, containing the indicated strains, on tomato (**A**), tobacco (**B**), and grapevine (**C**) leaves, on carnation (**D**) and gerbera (**E**) petals, and on tomato (**F**) and grape (**G**) fruits. Pictures were taken at the indicated day post inoculation (dpi). Inoculations were done either on intact plant material (without wound) or after wounding with a needle (with wound). Grapes were inoculated at the wound left by the detachment of the petiole.

The virulence of Δ*bcpmt* mutants was assayed in two ways, by quantifying the percentage of inoculations able to initiate infection on various hosts, and by determining the rate of expansion of the infection for successful inoculations (Table 1, Figure 8). The results indicated that all three Δ*bcpmt* mutant strains exhibit a reduction in their virulence when inoculated on intact plant tissue, as compared to the wild type. The percentage of inoculations producing infections was lower than the wild type for every mutant. Especially significant was the case of Δ*bcpmt2*, which was unable to infect intact leaves, petals, or fruits of any plant tested. Also noteworthy was the experiment with intact grapevine leaves, which were not infected by any of the three mutants (Table 1, Figure 8). The number of successful inoculations was also lower, in comparison to the wild type, for Δ*bcpmt1* and Δ*bcpmt4*, especially the latter, with a percentage of inoculations giving rise to expanding infections never higher than 35%. The percentage of successful inoculations with Δ*bcpmt1* and Δ*bcpmt4* were also calculated with gerbera and carnation petals (not shown), and were in the same range as those with tobacco and tomato.

**Table 1:** Virulence of the Δ*bcpmt* mutants on various hosts.

Strain	Tomato			Tobacco			Grapevine		
	Infection rate^a^ (%)		Lesion growth rate^b^ (mm/day)	Infection rate^a^ (%)		Lesion growth rate^b^ (mm/day)	Infection rate^a^ (%)		Lesion growth rate^b^ (mm/day)
	w/o wound	with wound		w/o wound	with wound		w/o wound	with wound	
B05.10	98	100	3.1±0.8	78	100	1.8±1.1	93	100	1.6±0.8
Δ*bcpmt1*	89	94	1.9±0.5*	69	97	1.4±0.7	0	83	0.7±0.3*
Δ*bcpmt2*	0	0	N/A	0	50	0.7±0.2*	0	0	N/A
Δ*bcpmt4*	34	79	1.6±0.6*	17	100	1.1±0.4*	0	60	0.4±0.2*

a Percentage of inoculations resulting in visible infections at 4 dpi (n ≥30). Leaves were wounded, where indicated, by puncturing with a needle. Inoculum consisted of 0.2 sq. mm agar cubes with mycelium.

b Growth rate of the radius of the infected area, calculated for successful inoculations (n ≥15).

* Asterisks indicate a statistically significant difference with the wild type in the same plant.

N/A: not applicable. w/o: without.

To analyze if these diminished capacities to produce infections are due to a reduced ability to penetrate the intact plant tissue, the same assay was repeated but this time wounds were made in the leaves with a sterile needle just below the agar plugs used as inoculum. The wounding had a stimulating effect on the infection rate for all the mutants and also for the wild type (Table 1), but the most impressive results were observed for Δ*bcpmt4*. Wounding allowed this mutant to produce infections in most of the inoculations for every plant tested including grapevine leaves. This is in good agreement with the fact that the BcPMT4 homologue in *U. maydis* has been proposed to be essential for penetration of maize leaves [24]. Recovery of the infection rate by wounding was also observed for petals of carnation and gerbera (not shown).

The speed of progression of the infections was also different for the three Δ*bcpmt* mutants as compared to the wild type (Table 1). Under all conditions tested, the average growth rate of the radius of the infected area was significantly smaller than the corresponding value in the wild-type strain. These differences were plant-dependent and varied in the range of 44–77% for Δ*bcpmt1*, 25–61% for Δ*bcpmt4*, and 39% for Δ*bcpmt2*, with respect to the growth rate of the wild type. This parameter was calculated for Δ*bcpmt2* only in tobacco leaves, the only plant that this mutant could infect. These values are not very different from the diminishment of growth rates in axenic culture caused by the mutation of *bcpmt* genes (Figure 2), leading to the conclusion that once the infection has been established, BcPMTs do not seem to play any especially significant role which is specific to the growth in planta, different from what they do in axenic culture. The main contribution of BcPMTs to the infection process seems to reside, therefore, in its ability to facilitate the attachment or penetration of the fungus on intact plant surfaces.

**Figure 9:**
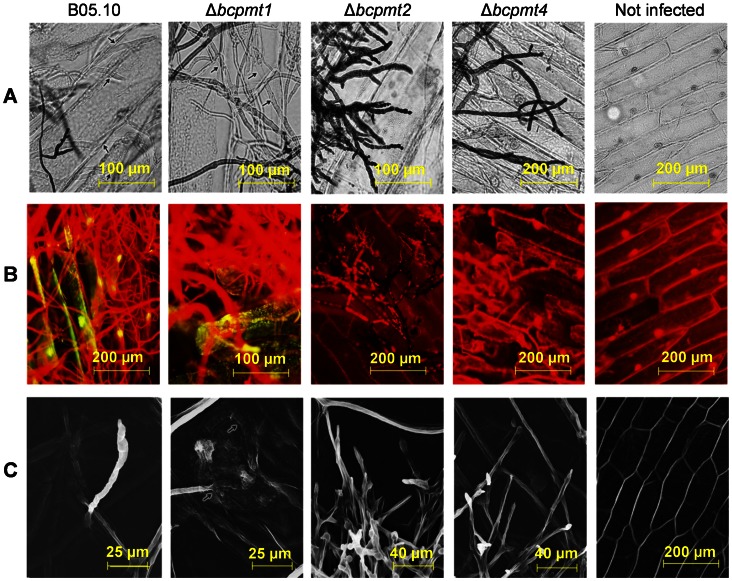
Penetration assays with the Δ*bcpmt* mutants on onion epidermis. Small pieces of mycelia from the three mutants and the wild type (B05.10) were placed on onion epidermis (inner surface up) and incubated for 1–4 days on the surface of water-agar. **A**) Samples stained with lactophenol trypan-blue. Arrows indicate unstained hyphae that have penetrated the epidermis. **B**) Fluorescence microscope observation of the same samples stained with lactophenol trypan-blue. Yellow autofluorescence is due to the release of phenolic compounds in the infected onion epidermal cells, and is an indirect indication of penetration. **C**) Observation under the scanning electron microscope. Arrows indicate the points where the mycelium has entered or is below the surface.

This hypothesis was further explored by performing penetration assays on onion epidermis inoculated with agar plugs coming from cultures of the three mutants, or the wild type. Figure 9A shows pictures of samples stained with lactophenol trypan-blue, which densely stains only those hyphae that have not penetrated the plant tissue. Weakly stained hyphae inside the onion cells could be observed for the wild type and for Δ*bcpmt1*, but not for Δ*bcpmt2 or* Δ*bcpmt4.* Similarly, Δ*bcpmt2* and Δ*bcpmt4* were also unable to stimulate autofluorescence in onion cells (Figure 9B), a phenomenon indicative of a successful infection [46]. Finally, examination of the infected tissues with a Scanning Electron Microscope (Figure 9C) revealed the presence of fungal penetration sites displaying macerated plant tissue in the case of the wild type and the Δ*bcpmt1* mutant, but these sites could not be found for the two other mutants which displayed, instead, plenty of fungal hyphae growing outside the onion cells.

**Figure 10:**
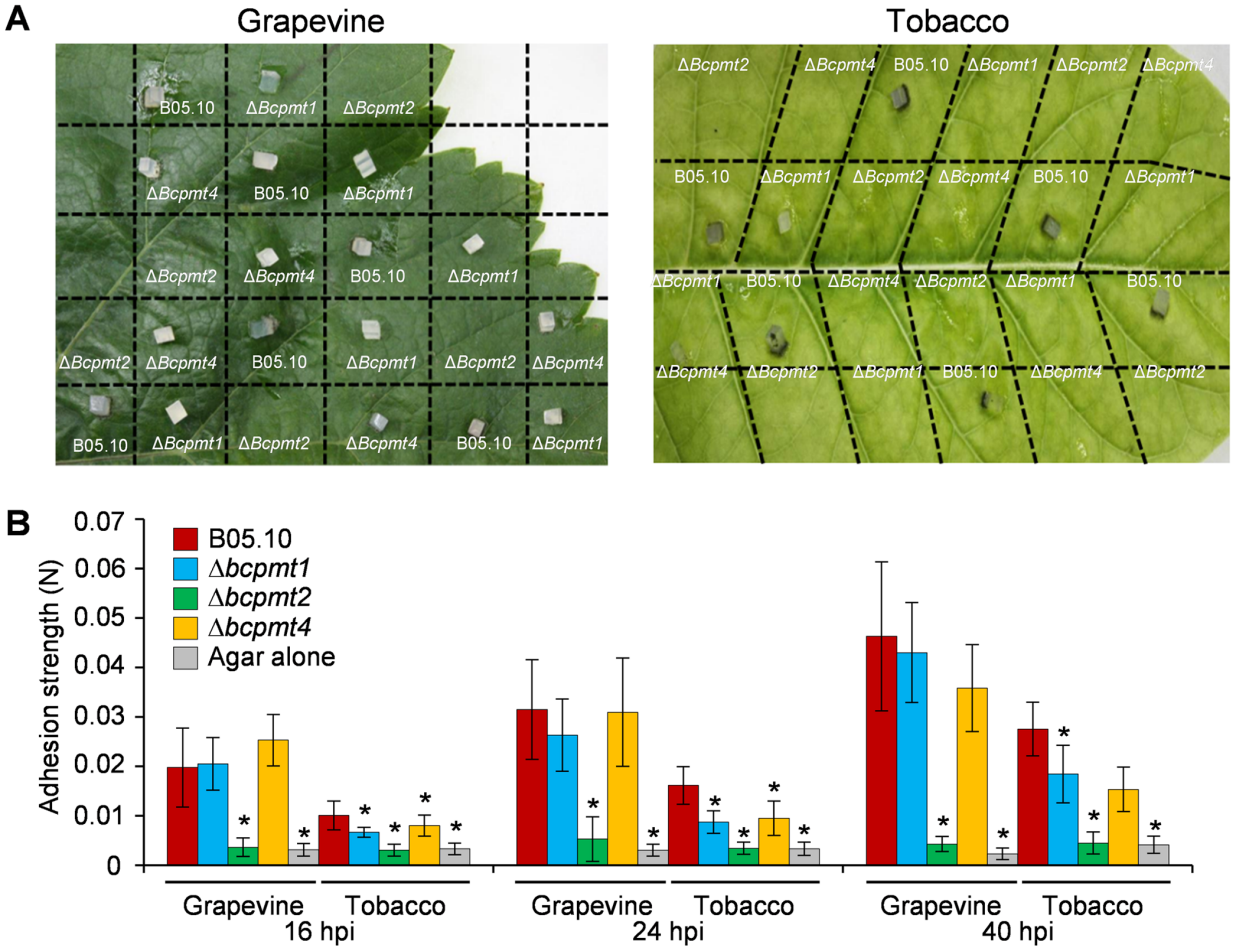
Δ*bcpmt2* is impaired in adhesion to plant surfaces. **A**) Grapevine and tobacco leaves, inoculated at random positions with mycelium plugs from the three Δ*bcpmt* mutants and the wild type (B05.10), were washed with water after 24-hours growth to remove non-attached plugs. **B**) Adhesion strength (mean values and standard deviation, n≥10) of agar plugs on grapevine and tobacco leaves at different times (hpi: hours after inoculation). Asterisks indicate a statistically significant difference with the wild type in the same conditions (p<0.05).

Another indirect test for the lack of penetration/adherence of Δ*bcpmt* mutants on intact plant surfaces was carried out by measuring the strength with which mycelial plugs adhere to tobacco or grapevine leaves, at different times after inoculation. In first place, we tested the resistance of plugs to be washed off from the leaf surface by water, one day after inoculation and before any symptom could be seen in the leaves. On grapevine leaves, only the Δ*bcpmt2* mutant could be removed by gentle agitation with water, while on tobacco leaves all Δ*bcpmt2* and Δ*bcpmt4* plugs were removed, as well as part of the Δ*bcpmt1* plugs (Figure 10A). The plugs with the wild type remained in the leaves in all occasions. The determination of the adherence strength, by pulling the agar plugs with a dynamometer, confirmed the lack of adherence of the plugs with Δ*bcpmt2*, and also showed that Δ*bcpmt1* and Δ*bcpmt4* tend to bind to the leaf surface with less strength than the wild type (Figure 10B).

We can summarize these results by saying that the three Δ*bcpmt* mutants are greatly hampered in virulence, not only because they grow *in planta* slower than the wild type, which also happens in axenic culture, but also because they experience difficulties in adhering to/penetrating the plant surface. It is interesting to note that the three Δ*bcpmt* mutants seem to differ among them, in regard to virulence, in a way similar to what has been observed for any other aspect of their phenotype. That is, Δ*bcpmt2* is the mutant with the strongest phenotype, Δ*bcpmt1* is the one most similar to the wild-type, and Δ*bcpmt4* presents intermediate features.

### Identification of Proteins Differentially *O*-glycosylated by Δ*bcpmt* Mutants

**Figure 11:**
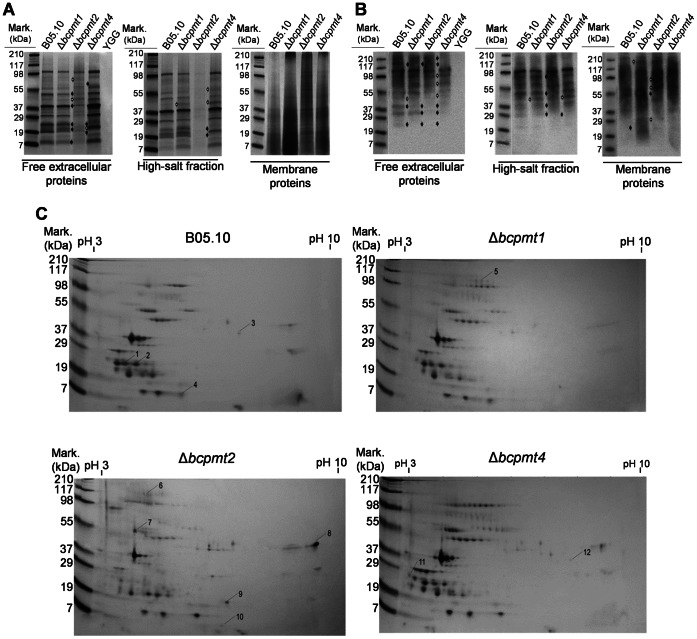
Patterns of protein secretion in the Δ*bcpmt* mutant strains. **A**) and **B**) SDS-PAGE gels of the secretory proteins produced by the three Δ*bcpmt* mutants and the wild type (B05.10). Proteins were either collected from the culture medium (Free extracellular proteins), extracted from intact mycelia with high-salt (High-salt fraction), or extracted from whole membranes (Membrane proteins). Proteins where either stained with Coomassie brilliant blue (A) or detected with Concanavalin A in lectin-blots (B). The control sample (YGG) shows that no proteins were obtained from the culture medium alone. White arrows point to bands that are new in the mutants and black arrows to bands that have disappeared, as compared to the wild type. **C**) Two dimensional electrophoresis of proteins isolated from the culture supernatants. Proteins in numbered spots were identified and are described in the text.

The changes in the *O*-glycosylation pattern introduced by the deletion of one of the *bcpmt* genes can result in an altered electrophoresis mobility of individual proteins, which may allow the identification of proteins serving as substrates of individual PMTs. This possibility was first explored by SDS-PAGE analysis of the proteins obtained from the extracellular medium, the high-salt fraction (proteins extracted with high salt from intact mycelium, presumably enriched in ECM proteins), and the cell membrane (Figure 11A). Initial trials (not shown), revealed that the amount of proteins that could be collected from the extracellular medium or by high-salt extraction was higher in cultures grown without shaking, probably because shaking greatly influences the pattern of growth (Figure 5D), so static liquid cultures were used. The results showed visible changes in the SDS-PAGE band pattern for extracellular and high-salt protein fractions from the three Δ*bcpmt* mutants, as compared to the wild type. These changes were more evident in case of Δ*bcpmt2* and Δ*bcpmt4*, which is consistent with the more extreme phenotypes described above for these two strains. The band pattern obtained for the high-salt fraction did not differ to a great extent from that obtained for extracellular proteins, so that this fraction seems like a continuation of the extracellular medium where proteins are simply trapped in the gel-like polysaccharide forming the ECM. Protein constituents of the ECM, predicted from results in Figures 7 and S4, may be more strongly attached to the polysaccharides and therefore not amenable to extraction with high salt.

The high abundance of glycosylated proteins in these protein preparations was shown by probing these protein preparations with Concanavalin-A in a lectin-blot. The pattern obtained (Figure 11B) shows, in first place, the vast number of secretion proteins that display sugars recognized by Concanavalin-A. This great number of glycosylated proteins has been predicted with *in silico* tools in the case of *O*-glycosylation [7]. Notably, the most intense band of about 38 kDa in the gels stained with Coomassie blue (Figure 11A), previously identified as the aspartic protease BcAP8 [47], did not produce an equally intense band in the lectin blot. This may be related with the fact that this protein is predicted to have only two N-glycosylation sites, by NetNGlyc (http://www.cbs.dtu.dk/services/NetNGlyc), and no *O*-glycosylation site, by NetOGlyc [7], and band intensities in the lectin-blot should relate to the amount of sugars and not to the amount of protein. In second place, despite the blurred bands typical of glycosylated proteins, this approach also resulted in clear differences in the band patterns for the wild type and the three mutants, indicating that individual PMTs have different, maybe overlapping, sets of substrate proteins.

In order to identify individual proteins with differential electrophoretic mobility, two-dimensional electrophoresis were carried out to analyze the proteins secreted to the culture media by the three Δ*bcpmt* mutants and the wild-type (Figure 11C). As we had previously observed [48], the complexity of the protein mixture in the secretome was relatively low for the four strains, only 65 spots were observed in the gel for the wild-type strain. The spot pattern observed in the Δ*bcpmt1* gel was the most similar to the wild type: 61 of 68 spots were also present in the wild-type strain, 7 new spots appeared and 4 disappeared. In the case of Δ*bcpmt2*, 48 of 79 spots were also in the wild-type gel, 31 new spots appeared and 17 disappeared. For the Δ*bcpmt4* sample, 63 of 101 spots were similar to wild type, 38 new spots appeared and only 2 disappeared. Intriguingly, the 2D gel for the Δ*bcpmt4* sample showed longer charge trains than the rest of the gels, a fact that may be in relation with differential glycosylation with charged sugars displaying sulfate or phosphate groups, previously described in related fungus such as *Trichoderma* [3], [49]. Charge trains as a result of multiple phosphorylations in intracellular proteins have a similar appearance [50]. The appearance/disappearance of spots is probably mainly a consequence of proteins changing mobility due to altered *O*-glycosylation, although other factors may also be relevant. For example, proteins with an altered glycosylation pattern may be incorrectly folded and may be retained in the secretion pathway. And the opposite could also be true, *O*-glycosilated proteins normally retained in the cell wall or ECM may end in the extracellular medium if they have an altered glycosylation pattern.

Twelve of the spots with a clear modified electrophoretic mobility (numbered in Figure 11C) were excised from the gels to identify the proteins in them by MALDI TOF/TOF. Spots 1, 2 and 11 were identified as BC1G_12374.1/BofuT4_P023950.1, a 187-aa hypothetical protein of unknown function similar to proteins described as IgE-binding proteins. This protein contains a region of 29 amino acids with more than 40% Ser/Thr residues, which is predicted to be hyper-*O*-glycosylated [7]. Interestingly, this protein seems to be affected both in the Δ*bcpmt2* and in the Δ*bcpmt4* mutants but in a different manner, several spots in the same charge train in the wild type gel (probably all BC1G_12374.1/BofuT4_P023950.1) simply disappear in Δ*bcpmt2* while in Δ*bcpmt4* they are still present but the charge train has been enlarged. The spot number 3 was identified as BC1G_08658.1/BofuT4_P123410.1, a 484-aa protein similar to Carboxypeptidase-A. This spot disappears in the Δ*bcpmt2* secretome, and its sequence contains a 19-aa region with a high Ser/Thr content. Spot number 4 shows a small shift to basic pI in Δ*bcpmt2* and Δ*bcpmt4* samples, and was identified as BcSpl1, a 137-aa cerato-platanin family protein required for full virulence and recently characterized by our group [51]. Surprisingly, no Ser/Thr-rich regions can be found in this protein and no *O*-glycosylation sites are predicted [7]. Spot number 5 is part of a charge train that appears only in Δ*bcpmt1* and was identified as BC1G_04151.1/BofuT4_P095270.1, a 672-aa protein similar to Glucoamylase which contains three Ser/Thr-rich regions [7]. This spot appears as part of a charge train around pH 5 that could be observed only in Δ*bcpmt1*, but has been previously identified at more acid pH [48]. Spot number 6 is in another charge train which was only observed in the Δ*bcpmt2* sample and was identified as BC1G_02021/BofuT4_P061670.1, a 628-aa protein similar to Glucose-methanol-choline oxidoreductases which contains two Ser/Thr-rich regions [7]. Spot number 7 is present only in the gel for Δ*bcpmt2* and was identified as BC1G_03070/BofuT4_P134040.1. This protein has been previously characterized as Aspartic protease 8 (BcAp8) [47], the most abundant secretome protein which forms the strongest spot in the gels [48] from which spot 7 seems to be a delayed fraction. Spot 8 was identified as Endopolygalacturonase 1 (BC1G_11143.1/BofuT4_P089370.1), a protein required for full virulence in *B. cinerea* [52]. This spot appeared in the gel for Δ*bcpmt2* at a pH more basic than we have previously observed in the wild type [48]. Bcpg1 displays four Ser/Thr-rich regions [7]. Spot number 9 was present only in the Δ*bcpmt2* gel and was identified as BC1G_08642/BofuT4_P123260.1, a 153-aa hypothetical protein of unknown function. Its sequence contains a 20-aa Ser/Thr-rich region [7]. The spot number 10 was only present in the gel for Δ*bcpmt2* and was identified as BofuT4_P024480.1, no corresponding protein was found in the sequenced genome of *B. cinerea* B05.10. This 121-aa protein has no known function and no similarities other than to its homologue in the closely related fungus *Sclerotinia sclerotiorum*. Despite having no predicted signal peptide, it has been previously found in the culture medium as an 11-kDa protein, named *ekdA*, that copurified with cutinase CutA [53], as well as in a previous proteomic study [48]. Its presence in the culture medium was explained as a leak from the cell interior [53], and this would agree with the fact that Δ*bcpmt2* is the mutant with the weakest cell wall. However, the fact that *ekdA* contains two Ser/Thr-rich regions would agree with extracellular location. Finally, the spot number 12 was observed only in the Δ*bcpmt4* sample and was identified as BC1G_10789.1/BofuT4_P064400.1, a 320-aa protein similar to alpha-L-arabinofuranosidases belonging family 62 of Glycosyl Hydrolases. This protein contains four Ser/Thr-rich regions [7].

Overall, ten different proteins have been identified with an altered electrophoretic mobility in the Δ*bcpmt* mutants. Almost all of them (9 out of 10) display in their sequence regions with a high Ser/Thr content (40% or more), usually considered as a key feature of regions to be subjected to *O*-glycosylation in the secretory pathway [5], [6].

## Discussion

In this work we have found that, similarly to other filamentous fungi, the *B. cinerea* genome contains three *pmt* genes, coding for representatives of each of the three PMT subfamilies. The three PMT proteins display the usual motifs and structure (transmembrane domains) typical of this kind of proteins, and show a near-constitutive expression. *pmt* genes in other systems are also expressed in all conditions examined with no significant variation. Such are the cases, for example, of the *pmt* genes from *C. neoformans* [21], which shown essentially no variation in the conditions assayed, and *A. nidulans* [33], [40], also showing constitutive expression for the gene in subfamily 2 and with a small decrease relative to culture age for genes in subfamilies 1 and 4. In the case of *S. cerevisiae*, regulation of *pmt* genes along the cell cycle has been reported, with maximal expression at the late G1 phase, and indeed binding sites for the SBF transcription factor involved in activation of transcription at the late G1 phase were encountered in the promoters of most *pmt* genes [10]. Despite these variations, *S. cerevisiae pmt* genes are also considered to have a high constitutive expression [10].

Knockout of the *pmt* genes in *B. cinerea* resulted in an array of defects on fungal growth, morphology, and virulence. These changes are most prominent for the Δ*bcpmt2* mutant, which grows very poorly, producing very compact colonies with thick hyphae displaying small swollen cells and more frequent ramifications. This phenotype reverts partially with the addition of osmotic stabilizers, pointing to a weakened cell wall as the main cause. Furthermore, Δ*bcpmt2* was also completely unable to produce ECM and to adhere to, or penetrate, intact plant surfaces. Its growth *in planta* could only be observed in wounded tobacco leaves, from all the plant material tested. All these findings indicate that BcPMT2 contributes in a great extent to the *O*-glycosylation of proteins which are important to maintain the structure of the cell wall and extracellular matrix, and, either solely as a consequence of this or because of the *O*-glycosylation of virulence-specific proteins, BcPMT2 is also essential for virulence. Deletion of the homologous gene in other filamentous fungi results always in similar strong phenotypes, even in lack of viability. Mutants for the *bcpmt2* homologues in *A. awamori* [16] and *A. nidulans* [40] are both viable but show similar growth defects and morphology as Δ*bcpmt2*, but the deletion of the corresponding gene in *Aspergillus fumigatus* [34], *Candida albicans* [37], and *U. maydis* [24] is lethal. Conflicting results have been reported for *S. pombe* [22], [23]. PMT2 seems to be, therefore, the PMT enzyme playing the most important role in fungal basic biology, and evidences point to a crucial role in the *O*-glycosylation of proteins important in maintaining the structure of the fungal cell wall and extracellular matrix.

The deletion of *bcpmt4* results in a phenotype that seems to be a weaker version of the Δ*bcpmt2* phenotype but with added extra features. In almost every aspect assayed, such as growth rate, appearance under the microscope, production of conidia, and formation of ECM, Δ*bcpmt4* seems to be half-way between the wild type and Δ*bcpmt2*. Moreover, some of these features were reverted by the addition of osmotic stabilizers, in the same way as Δ*bcpmt2*, also pointing to a damaged cell wall in this mutant. This kind of phenotype, related with a weaker cell-wall, was also found in *pmt4* mutants of *A. nidulans* [25], [33], *A. fumigatus* [34], *C. neoformans* 2844 [54], and to a minor extent in *U. maydis* [24], whose only symptom of a weakened cell-wall is an increased sensitivity to SDS. This indicates that protein substrates of BcPMT4 include cell wall proteins, although their quantity or relevance in cell wall structure is not as high as in the case of BcPMT2.

BcPMT4, on the other hand, seems to play a specific role in virulence. Δ*bcpmt4* is highly impaired in penetration (Figures 8 and 9, Table 1), similarly to what has been observed for the *pmt4* mutant of *U. maydis* [24]. Although the same impairment was observed in Δ*bcpmt2*, the effect seems to be much more specific in Δ*bcpmt4*. As compared with Δ*bcpmt2*, Δ*bcpmt4* displays a general fitness phenotype in axenic culture (growth rates, sporulation, etc.) more similar to the wild type. However, its penetration ability, especially in grapevine leaves, is virtually nonexistent. These findings point to a role of PMT4 in the *O*-glycosylation of proteins with specific roles in penetrating the plant surface, and in this respect it is interesting that in *U. maydis* the *pmt4* mutant produces a reduced number of appressoria, and those produced are not capable to carry on to penetrate the cuticle [24].

BcPMT1 appears to be the PMT protein contributing the less to the fitness of *B. cinerea*. Although the Δ*bcpmt1* mutant also showed a reduction in the growth rate not very different from that obtained for Δ*bcpmt4*, the appearance of Δ*bcpmt1* mycelia and colonies under the microscope (Figure 6) is very similar to the wild type. Regarding virulence, PMT1 also seems to contribute to a lesser extent, and a significant decrease was only seen for the penetration of grapevine leaves, but not tomato or tobacco leaves and onion epidermis.

One important aspect of Δ*bcpmt* mutants is its decreased ability to generate ECM, and the probably related lower capacity to retain water (Figure 7). Although ECM was most affected by the deletion of *bcpmt2*, the mutation of the other two genes also has the consequence of producing a somehow weaker matrix, which is more easily removed when the culture is shaken. *O*-glycosylated proteins are therefore expected as crucial components of ECM, and these would be substrates mainly of BcPMT2 but also of BcPMT4. The existence of these structural proteins in ECM is also predicted by the fact that ECM in the wild-type strain becomes disorganized to some extent when whole cultures are treated with proteinase K (Figure S4). The deletion of the *pmt4* gene in the human pathogen *Cryptococcus neoformans* also results in about 50% reduction in the size of the polysaccharide capsule [21], a virulence factor for this organism, although the contribution of PMT2 could not be assessed because the corresponding mutant was not viable. It is tempting to make ECM, or the lack of it, responsible for the decreased adherence to plant surfaces observed for the three mutants (Figure 10), since the degree of adherence seems to be related to the amount of ECM. However, given the pleiotropic nature of the *bcpmt* mutations, the generation of mutants specifically affected in ECM would be necessary to test this hypothesis.

As has been discussed before [33], the phenotypes observed for the three *pmt* mutants in *B. cinerea* and in other fungi [24], [25], [33], [34], [40] do no easily agree with the dimerization of PMT proteins described in *S. cerevisiae* [15]. If the heterodimerization of *S. cerevisiae* PMT1 and PMT2 subfamily members, and the homodimerization of PMT4, were also true for their fungal homologues, and if this dimerization were necessary for the PMT proteins to have protein mannosylation activity, then one would expect similar phenotypes for *pmt1* and *pmt2* mutants. Since this is clearly not the case, one of the two above conditions is not true for these organisms. The usually stronger phenotype, often lethal, of the *pmt2* mutants would be compatible with the existence of PMT1/PMT2 and PMT2/PMT4 heterodimers, but this possibility has never been explored experimentally in fungi, to our knowledge.

We have identified several secretome proteins with altered electrophoretic mobility in the *pmt* mutants, which constitute potential new PMTs substrates. Almost all of them were predicted previously to be *O*-glycosylated [7]. For 2 of these proteins, as well as for several more unidentified spots in 2D gels, the electrophoretic mobility was altered in more than one of the *pmt* mutants. This may be a consequence of PMT heterodimerization as has been discussed above, the *O*-glycosylation of proteins which are substrates of a given hetero-dimer would be affected in the two corresponding mutants, but may also be possible if individual proteins can serve as substrates to more than one PMT or PMT homo/hetero-dimer. The expression in *B. cinerea pmt* mutants of short Ser/Thr-rich regions predicted to be hyper-*O*-glycosilated [7], bound to reporter proteins, may help elucidate this matter in the future.

One important implication of this work, given the great impact that the *pmt* mutations have on virulence and viability, and given the fact that plants do not have PMTs, is the possibility of using these enzymes as targets in the design of novel control strategies against *B. cinerea*. The fact that the deletion of only one *pmt* gene, out of three, can render the fungus non-virulent suggests that PMT inhibitors may be useful in the fight against this plant pathogen, even if inhibition is not complete.

## Materials and Methods

### Organisms and Culture Conditions

*B. cinerea* strain B05.10 [55] was used as wild-type and control strain. Fungal strains were kept as conidia or mycelium (in agar plugs, for non-conidiating strains) in 15% glycerol at −80°C for long time storage, or in silica gel at 4°C for routine use [56]. Tomato-agar plates (25% tomato fruit extract, 2% agar, pH 5.5) were inoculated with silica stocks or agar plugs to obtain conidia [57]. Conidia were routinely quantified at 600 nm with a spectrophotometer. The relation between Abs_600_ and number of conidia per mL was calculated for our spectrophotometer with the aid of a hemocytometer. Sclerotia viability was assayed by washing them 3 times for 10 min with water, 3 min with 1% NaClO, again 3×10 min with water, and finally inoculating them onto YGG plates. Routine culture or growth tests were carried out in MEA (1% Malt Extract from Pronadisa, Spain), SH (600 mM Sucrose, 5 mM HEPES pH 6.5, 1 mM NH_4_H_2_PO_4_), YGG (0.5% Yeast Extract from Pronadisa, 100 mM Glucose, 0.3% Gamborg’s B5 from Duchefa Biochemie), PDA (3.9% Potato dextrose agar from Duchefa Biochemie), GB5 (0.3% Gamborg’s B5, 10 mM Glucose), and MB (0.3% Gamborg’s B5, 10 mM Glucosa, 10 mM KH_2_PO_4_, 10 mM MES pH 5.5). All media were supplemented with 1.5% agar when necessary.

### Bioinformatic and Statistical Analysis

Sequence alignments and phylogenetic analyses were made with CLUSTALW2 [58], using the default settings. Transmembrane regions were predicted with TMHMM 2.0 [27], PredictProtein [28], [29], and Phobius [30]. Transmembrane regions for whole PMT families (Figure S1) were represented by calculating the average start and end positions of each region, as predicted by Phobius, in the alignment (Figure S2). Conserved sequence motifs were searched for with InterPro Scan (http://www.ebi.ac.uk/Tools/pfa/iprscan). Average hydropathy profiles for whole PMT families (Figure S1) were calculated by manually determining the average Kyte-Doolittle index for all the residues in a given alignment position, in first place, and then deriving from them the average index over windows of 15 alignment positions. Statistical analysis was carried out with SPSS 17 (IBM). Statistical significance tests used were either the T-test, in those cases with a normal distribution (analyzed with the Kolmogorov-Smirnov test), or the Mann-Whitney test, if sample distribution was not normal.

### Quantitative Real Time PCR

Mycelia for RNA extraction were prepared as explained elsewhere [47]. Total RNA from *B. cinerea* and *B. cinerea*-infected tomato plants was isolated with the RNeasy plant mini kit (Qiagen). Contaminant genomic DNA was eliminated by treatment with RNase-free DNaseI. Amplification was carried out in an iCycler iQ Real-Time PCR system (Bio-Rad), with the Bio-Rad iQ SYBR Green supermix and the primers listed in Table S2. The amplification of a single fragment was verified for every PCR reaction by running the final product on a 12% polyacrylamide gel electrophoresis. The relative mRNA amounts were calculated by the ΔΔCt method from the mean of three independent determinations of the threshold cycle [59], [60]. Deviation from the mean was calculated from the standard deviation (SD) in the ΔΔCt value, using the expression 2^(ΔΔCt ± SD)^.

### *bcpmt* Gene Replacements

Recombinant DNA methods were performed as described by Sambrook and Russell [61]. Primers (Table S2) were from Invitrogen (Paisley, Scotland). Genomic DNA from *B. cinerea* was extracted using a method previously developed in our laboratory [62]. The gene replacement strategy is outlined in Figure S3. The replacement cassettes were constructed by DJ-PCR following a method previously described [63], [64], with some modifications. The hygromycin resistance cassette (GenBank accession AJ439603) was amplified in all cases with Phusion DNA polymerase (Finnzymes, Keilaranta, Finland), and the two flanking regions homologous to the target gene (approximately 500 bp) were amplified with Taq DNA polymerase (GenScript, Picataway, NJ, USA) from *B. cinerea* B05.10 genomic DNA. Equimolar amounts of the three fragments were fused by PCR, and the final constructs were amplified with the corresponding nested primers (Figure S3), using 2 µl of each fusion PCR as template. The resultant gene replacement cassettes were purified from agarose gels with Zymoclean Gel DNA Recovery Kit (Zymo Research Corporation, Irvine, U.S.A.) and checked by digestion with restriction enzymes. *B. cinerea* transformations were carried out as described elsewhere [65], [66] and transformants were then purified by single-conidia isolation on hygromycin-containing plates to ensure homokaryosis. Homokaryotic transformants were analyzed by Southern-blot and by PCR (Figure S3) to ensure the correct disruption of the *bcpmt* gene and the absence of additional copies of the transforming DNA. Southern blots were carried out with digoxigenin labeled probes using the DIG DNA Labeling and Detection kit (Roche, Basel, Switzerland).

### Microscopy

Staining with India ink was carried out by incubating mycelia with the dye for 5 min, and samples were then directly examined with the microscope. Staining with CW was carried out also for 5 min with CW stain solution (0.05% CW, 7.5% KOH), samples were then washed twice with 15% KOH and finally observed under an Olympus BX-50 fluorescence microscope equipped with a U-MWIB filter. For scanning electron microscopy (SEM), mycelia were grown from agar plugs over MF-Millipore filters (0.22 µm) layered on MB plates. Two days after inoculation filters were collected, fixed with 25% glutaraldehyde vapors on a vacuum hood for 24 hours, thoroughly dried with silica-gel under vacuum for 5 days, coated with gold, and observed in a JEOL JSM-6300 scanning electron microscope.

### Pathogenicity Tests

Since the Δ*bcpmt* mutants produce little or no conidia, the indicated plant material (detached leaves, petals, or fruits) was inoculated with mycelia in agar plugs (0.2-cm YGG-agar cubes) and 20 µl of sterile water were added over each plug to facilitate adherence. The infected plant material was incubated at 22°C under conditions of high humidity on water-soaked filter paper in closed containers, and lesions at different time-points were photographed. At least 30 replicates were performed in every case. Infections were evaluated visually and, only for tomato, tobacco and grapevine leaves, quantitative results are presented as the rate of progression of lesion size (radius in mm day^−1^), calculated for each individual infection from three measures taken at different days. For this purpose, the shape of the lesions was approximated to an ellipse for which the two radii were measured, and lesion sizes were calculated as the geometrical mean of these two radii, that is, as the radius of a hypothetical circle with the same area as the ellipse.

The strength with which the agar plugs (5-mm agar cubes) adhere to plant surfaces was measured by slowly pulling the agar plug with a dynamometer (Ventus, 0.1 N) attached to a camera stand, with the aid of a needle curved to resemble a hook attached to the plug. The pulling force displayed by the dynamometer at the time the plug detached from the leaf was recorded for a minimum of 10 replicates.

### Onion Epidermis Penetration Assays

Small pieces of onion epidermis were cut and placed on slides with the inner face up. A small piece of mycelium grown for 4 days in liquid medium was placed on it and one drop of YGG-broth was added. Samples were incubated in a humid chamber until mycelia was clearly growing (1 day for the wild type and 2–4 days for the mutants) and then stained with 0.05% trypan-blue in lactophenol for 5 min. After removing the excess dye by washing with water, the epidermis was examined under visible or UV (U-MWIB filter) light with the fluorescence microscope. SEM images were obtained as explained above, but in this case the fungus was grown from agar plugs over onion epidermis and the plugs were removed before drying the samples.

### Preparation of Protein Samples

In order to obtain the various protein fractions, agar plugs containing young mycelium were inoculated in plates with 20 ml of YGG-broth, approximately 5–20 plugs per plate depending on strain, and grown for 4 days without shaking. Culture media were then separated from mycelia by filtration through several layers of filter paper. Extracellular proteins were precipitated from the filtrate with methanol-chloroform according to Wessel and Flugge [67] and stored dry at −20°C until use, in several identical aliquots. The amount of proteins obtained was estimated by loading one of the aliquots to a SDS-PAGE gel along with known quantities of bovine serum albumin, as described before [48]. Typically, from one plate with 20 ml of culture medium 10–20 µg of proteins were obtained for Δ*bcpmt2* and 70–100 µg for the rest of the strains. In order to obtain proteins weakly bound to the extracellular matrix or the cell wall (referred to high-salt fraction), the mycelia retained in the filters were washed twice for 15 min with cold water under slow agitation and then extracted with high salt, by incubating with 300 mM NaCl (approximately the same volume as the mycelium) for 20 minutes under slow agitation. After centrifugation at 1000 *g* for 6 minutes, the proteins in the supernatant were precipitated with methanol–chloroform and quantified as explained above. In order to prepare proteins from cell membranes, the same mycelium samples were washed with cold water again and then a portion of approximately 0.2 g was homogenized in a FastPrep-24 MP beadbeater with 400 µl of cold Lysis Buffer (75 mM Tris-HCl pH7, 1.5 mM EDTA, 1 M Sucrose, and Complete Protease Inhibitor from Roche) and 0.5 mm glass beads at 60 m s^−1^ for 1 min. The tube was then punctured at the bottom with a needle and briefly centrifuged inside an empty microcentrifuge tube. The resulting filtrate was first centrifuged for 15 min at 3000 *g* in the cold (4°C), and the supernatant was re-centrifuged for 1 h at 145 000 *g*, also at 4°C. The pellet containing the membrane fraction was resuspended in SDS-PAGE sample buffer and quantified as explained above. All protein preparations were analyzed by SDS-PAGE on Any-KD Mini-PROTEAN TGX precast gels (Bio-Rad), and the gels were stained with colloidal Coomassie brilliant blue [68].

### Lectin Blots

Equal amounts of protein were loaded to Any-KD Mini-PROTEAN TGX precast gels (Bio-Rad) and then transferred to Immobilon PVDF membranes (Millipore). The membranes were then processed as explained elsewhere [69] using 1 µg µl^−1^ biotinylated concanavalin-A (Sigma C2272) to detect glycosylated proteins and 0.5 µg ml^−1^ Streptavidin-Peroxidase (Sigma S5512) to detect the biotinylated concanavalin. Final detection of the peroxidase was carried out with 400 µl of Immobilon Western Chemiluminescent HRP substrate (Millipore) using the Gel Doc XR+ System (Bio-Rad).

### 2-D Electrophoresis and Protein Identification

All reagents and equipment for 2-D electrophoresis were from Bio-Rad and were used according to the manufacturer’s instructions. Dry protein samples were dissolved in the appropriate volume of ReadyPrep sequential extraction Reagent 2 to achieve 10 µg of total protein in 125 µl. This amount of protein was subjected to 2D electrophoresis as explained before [48] except that the pH range of the IPG strips was 3–10 and Any-KD Mini-PROTEAN TGX precast gels (Bio-Rad) were used for the second dimension. Protein spots were excised manually from stained gels and sent to the CNB Proteomics Facility (Centro Nacional de Biotecnología, Madrid, Spain) to be processed by MALDI TOF/TOF analysis as described earlier [70]. Protein identification was done with MASCOT software v.2.2.04 (Matrix Science, London, UK), searching the non-redundant NCBI protein database NCBInr_20120720 (19248190 sequences; 6604124933 residues) with the following search parameters: enzyme, trypsin; allowed missed cleavages, 1; carbamidomethyl cystein as fixed modification by the treatment with iodoacetamide; variable modifications, oxidation of methionine; mass tolerance was set to ±50 ppm for precursors and to ±0.3 Da for MS/MS fragment ions. The confidence interval for protein identification was set to ≥95% (p<0.05) and only peptides with an individual ion score above the identity threshold were considered correctly identified.

## Supporting Information

Figure S1 **Putative topology of** ***B. cinerea*** **PMTs.** The hydrophobicity of each BcPMT (thick line), calculated with the Kyte-Doolittle scale and a window of 15 residues, is plotted alongside the average hydrophobicity of the corresponding PMT subfamily (thin line) derived from the alignment (Figure S2). Black lines below plot indicate the transmembrane regions predicted for the alignment, and those corresponding to the 7 transmembrane domains of *S. cerevisiae* PMTs are marked with roman numerals (question marks indicate that it is not clear which one of the two regions marked as VII is the last transmembrane domain in *S. cerevisiae* [14]). PMT domains (grey boxes) and MIR subdomains (white boxes) detected by InterProScan are also marked.
(TIF)

Figure S2 **Alignment of fungal PMTs.** Proteins were aligned by PRALINE, making use of the transmembrane regions predicted by PHOBIUS (on green background). The regions homologous to the 7 experimentally-determined transmembrane domains of *S. cerevisiae* Pmt1p are marked with roman numerals. Question marks indicate that it is not clear which region is the last transmembrane domain of Pmt1p. The catalytically-important ER-luminal loop 1 (red boxes), loop 5 (blue boxes), and DE-motif (yellow boxes) are also shown in the alignments. First two letters in protein names indicate the fungus it comes from (Sc: *Saccharomyces cerevisiae*; Ca: *Candida albicans*; Sp: *Schizosaccharomyces pombe*; Um: *Ustilago maydis*; Cn: *Cryptococcus neoformans*; Bc: *Botrytis cinerea*; An: *Aspergillus nidulans*; Af: *Aspergillus fumigatus*). Accession numbers can be found in Table S1.
(PDF)

Figure S3 **Generation of the three** ***bcpmt*** **knockout mutants. A**) Strategy used to generate the three Δ*bcpmts* knock-out mutants. The DNA constructs used in the transformation, shown below each *bcpmt* gene, contain the hygromycin resistance cassette (Hyg-R) flanked by two regions of the target gene. The position of all primers used to generate the constructs and to check the transformants are indicated (arrows). **B**) PCR and Southern-blot analysis carried out to check the mutants. Primers used in the PCRs (left panels) are indicated below each picture. Southern-blots were carried out with the indicated genomic DNAs and restriction enzymes, and with the probes indicated in A.
(TIF)

Figure S4 **Partial degradation of the extracellular matrix by treatment with proteinase K.** Mycelia were grown in liquid YGG medium without shaking, transferred to 0.5X phosphate buffer saline and incubated with 0.4 mg ml^−1^ proteinase K for 4 hours before staining with India ink. Controls received no proteinase K.
(TIF)

Table S1 **Protein sequences used in this work.** Protein sequences used in the comparative studies displayed in Figures 1 and S2.
(PDF)

Table S2 **Oligonucleotides used in this study.**
(PDF)

## References

[1] Lehle L, Strahl S, Tanner W (2006) Protein glycosylation, conserved from yeast to man: a model organism helps elucidate congenital human diseases. Angew Chem Int Ed Engl 45: 6802–6818. 17024709 10.1002/anie.200601645

[2] Burda P, Aebi M (1999) The dolichol pathway of *N*-linked glycosylation. Biochim Biophys Acta 1426: 239–257. 9878760 10.1016/s0304-4165(98)00127-5

[3] Goto M (2007) Protein *O*-glycosylation in fungi: diverse structures and multiple functions. Biosci Biotechnol Biochem 71: 1415–1427. 17587671 10.1271/bbb.70080

[4] Julenius K, Molgaard A, Gupta R, Brunak S (2005) Prediction, conservation analysis, and structural characterization of mammalian mucin-type *O*-glycosylation sites. Glycobiology 15: 153–164. 15385431 10.1093/glycob/cwh151

[5] Hutzler J, Schmid M, Bernard T, Henrissat B, Strahl S (2007) Membrane association is a determinant for substrate recognition by PMT4 protein *O*-mannosyltransferases. Proc Natl Acad Sci U S A 104: 7827–7832. 17470820 10.1073/pnas.0700374104 PMC1876532

[6] Fernández-Álvarez A, Marín-Menguiano M, Lanver D, Jiménez-Martín A, Elías-Villalobos A, et al (2012) Identification of *O*-mannosylated Virulence Factors in *Ustilago maydis* . PLoS Pathog 8: e1002563. 22416226 10.1371/journal.ppat.1002563 PMC3295589

[7] González M, Brito N, González C (2012) High abundance of Serine/Threonine-rich regions predicted to be hyper-*O*-glycosylated in the extracellular proteins coded by eight fungal genomes. BMC Microbiol 12: 213. 22994653 10.1186/1471-2180-12-213 PMC3579731

[8] Gentzsch M, Tanner W (1996) The PMT gene family: protein *O*-glycosylation in *Saccharomyces cerevisiae* is vital. EMBO J 15: 5752–5759. 8918452 PMC452322

[9] Lussier M, Sdicu AM, Bussey H (1999) The KTR and MNN1 mannosyltransferase families of *Saccharomyces cerevisiae* . Biochim Biophys Acta 1426: 323–334. 9878809 10.1016/s0304-4165(98)00133-0

[10] Strahl-Bolsinger S, Gentzsch M, Tanner W (1999) Protein *O*-mannosylation. Biochim Biophys Acta 1426: 297–307. 9878797 10.1016/s0304-4165(98)00131-7

[11] Lommel M, Strahl S (2009) Protein *O*-mannosylation: conserved from bacteria to humans. Glycobiology 19: 816–828. 19429925 10.1093/glycob/cwp066

[12] Girrbach V, Zeller T, Priesmeier M, Strahl-Bolsinger S (2000) Structure-function analysis of the dolichyl phosphate-mannose: protein *O*-mannosyltransferase ScPmt1p. J Biol Chem 275: 19288–19296. 10764776 10.1074/jbc.M001771200

[13] Willer T, Amselgruber W, Deutzmann R, Strahl S (2002) Characterization of POMT2, a novel member of the PMT protein *O*-mannosyltransferase family specifically localized to the acrosome of mammalian spermatids. Glycobiology 12: 771–783. 12460945 10.1093/glycob/cwf086

[14] Strahl-Bolsinger S, Scheinost A (1999) Transmembrane topology of Pmt1p, a member of an evolutionarily conserved family of protein *O*-mannosyltransferases. J Biol Chem 274: 9068–9075. 10085156 10.1074/jbc.274.13.9068

[15] Girrbach V, Strahl S (2003) Members of the evolutionarily conserved PMT family of protein *O*-mannosyltransferases form distinct protein complexes among themselves. J Biol Chem 278: 12554–12562. 12551906 10.1074/jbc.M212582200

[16] Oka T, Sameshima Y, Koga T, Kim H, Goto M, et al (2005) Protein *O*-mannosyltransferase A of *Aspergillus awamori* is involved in *O*-mannosylation of glucoamylase I. Microbiology. 151: 3657–3667. 10.1099/mic.0.28088-0 16272387

[17] Timpel C, Strahl-Bolsinger S, Ziegelbauer K, Ernst JF (1998) Multiple functions of Pmt1p-mediated protein *O*-mannosylation in the fungal pathogen *Candida albicans* . J Biol Chem 273: 20837–20846. 9694829 10.1074/jbc.273.33.20837

[18] Sanders SL, Gentzsch M, Tanner W, Herskowitz I (1999) *O*-Glycosylation of Axl2/Bud10p by Pmt4p is required for its stability, localization, and function in daughter cells. J Cell Biol 145: 1177–1188. 10366591 10.1083/jcb.145.6.1177 PMC2133149

[19] Lommel M, Bagnat M, Strahl S (2004) Aberrant processing of the WSC family and Mid2p cell surface sensors results in cell death of *Saccharomyces cerevisiae O*-mannosylation mutants. Mol Cell Biol 24: 46–57. 14673142 10.1128/MCB.24.1.46-57.2004 PMC303345

[20] Hirayama H, Fujita M, Yoko-o T, Jigami Y (2008) *O*-mannosylation is required for degradation of the endoplasmic reticulum-associated degradation substrate Gas1*p via the ubiquitin/proteasome pathway in *Saccharomyces cerevisiae* . J Biochem 143: 555–567. 18182384 10.1093/jb/mvm249

[21] Willger SD, Ernst JF, Alspaugh JA, Lengeler KB (2009) Characterization of the PMT gene family in *Cryptococcus neoformans* . PLoS One 4: e6321. 19633715 10.1371/journal.pone.0006321 PMC2711527

[22] Willer T, Brandl M, Sipiczki M, Strahl S (2005) Protein *O*-mannosylation is crucial for cell wall integrity, septation and viability in fission yeast. Mol Microbiol 57: 156–170. 15948957 10.1111/j.1365-2958.2005.04692.x

[23] Tanaka N, Fujita Y, Suzuki S, Morishita M, Giga-Hama Y, et al (2005) Characterization of *O*-mannosyltransferase family in *Schizosaccharomyces pombe* . Biochem Biophys Res Commun 330: 813–820. 15809069 10.1016/j.bbrc.2005.03.033

[24] Fernández-Álvarez A, Elías-Villalobos A, Ibeas JI (2009) The *O*-mannosyltransferase PMT4 is essential for normal appressorium formation and penetration in *Ustilago maydis* . Plant Cell 21: 3397–3412. 19880800 10.1105/tpc.109.065839 PMC2782298

[25] Kriangkripipat T, Momany M (2009) *Aspergillus nidulans* protein *O*-mannosyltransferases play roles in cell wall integrity and developmental patterning. Eukaryot Cell 8: 1475–1485. 19666781 10.1128/EC.00040-09 PMC2756865

[26] Espino JJ, Brito N, Noda J, González C (2005) *Botrytis cinerea* endo-ß-1,4-glucanase Cel5A is expressed during infection but is not required for pathogenesis. Physiol Mol Plant Pathol 66: 213–221.

[27] Krogh A, Larsson B, von Heijne G, Sonnhammer ELL (2001) Predicting transmembrane protein topology with a hidden Markov model: application to complete genomes. J Mol Biol 305: 567–580. 11152613 10.1006/jmbi.2000.4315

[28] Rost B, Sander C, Casadio R, Fariselli P (2008) Transmembrane helices predicted at 95% accuracy. Protein Sci 4: 521–533. 10.1002/pro.5560040318 PMC2143072 7795533

[29] Rost B, Yachdav G, Liu J (2004) The predictProtein server. Nucl Acids Res 32: W321–W326. 15215403 10.1093/nar/gkh377 PMC441515

[30] Käll L, Krogh A, Sonnhammer EL (2007) Advantages of combined transmembrane topology and signal peptide prediction - the Phobius web server. Nucleic Acids Res 35: W429–W432. 17483518 10.1093/nar/gkm256 PMC1933244

[31] Illergård K, Ardell DH, Elofsson A (2009) Structure is three to ten times more conserved than sequence - A study of structural response in protein cores. Proteins 77: 499–508. 19507241 10.1002/prot.22458

[32] Lommel M, Schott A, Jank T, Hofmann V, Strahl S (2011) A conserved acidic motif is crucial for enzymatic activity of protein *O*-mannosyltransferases. J Biol Chem 286: 39768–39775. 21956107 10.1074/jbc.M111.281196 PMC3220539

[33] Goto M, Harada Y, Oka T, Matsumoto S, Takegawa K, et al (2009) Protein *O*-mannosyltransferases B and C support hyphal development and differentiation in *Aspergillus nidulans* . Eukaryot Cell 8: 1465–1474. 19648468 10.1128/EC.00371-08 PMC2756868

[34] Mouyna I, Kniemeyer O, Jank T, Loussert C, Mellado E, et al (2010) Members of protein *O*-mannosyltransferase family in *Aspergillus fumigatus* differentially affect growth, morphogenesis and viability. Mol Microbiol 76: 1205–1221. 20398215 10.1111/j.1365-2958.2010.07164.x

[35] Schumacher J, Tudzynski P (2012) Morphogenesis and infection in *Botrytis cinerea*. In: Pérez-Martín J, Di Pietro A, editors. Morphogenesis and pathogenicity in fungi. Berlin/Heidelberg: Springer. 225–241.

[36] Liu W, Soulié MC, Perrino C, Fillinger S (2011) The osmosensing signal transduction pathway from *Botrytis cinerea* regulates cell wall integrity and MAP kinase pathways control melanin biosynthesis with influence of light. Fungal Genet Biol 48: 377–387. 21176789 10.1016/j.fgb.2010.12.004

[37] Prill SKH, Klinkert B, Timpel C, Gale CA, Schroppel K, et al (2005) PMT family of *Candida albicans*: five protein mannosyltransferase isoforms affect growth, morphogenesis and antifungal resistance. Mol Microbiol 55: 546–560. 15659169 10.1111/j.1365-2958.2004.04401.x

[38] Bowman SM, Free SJ (2006) The structure and synthesis of the fungal cell wall. Bioessays 28: 799–808. 16927300 10.1002/bies.20441

[39] Doss RP (1999) Composition and enzymatic activity of the extracellular matrix secreted by germlings of *Botrytis cinerea* . Appl Environ Microbiol 65: 404–408. 9925560 10.1128/aem.65.2.404-408.1999 PMC91039

[40] Oka T, Hamaguchi T, Sameshima Y, Goto M, Furukawa K (2004) Molecular characterization of protein *O*-mannosyltransferase and its involvement in cell-wall synthesis in *Aspergillus nidulans* . Microbiology 150: 1973–1982. 15184583 10.1099/mic.0.27005-0

[41] Fang W, Ding W, Wang B, Zhou H, Ouyang H, et al (2010) Reduced expression of the *O*-mannosyltransferase 2 (AfPmt2) leads to deficient cell wall and abnormal polarity in *Aspergillus fumigatus* . Glycobiology 20: 542–552. 20053626 10.1093/glycob/cwp206

[42] Bowman SM, Piwowar A, Ciocca M, Free SJ (2005) Mannosyltransferase is required for cell wall biosynthesis, morphology and control of asexual development in *Neurospora crassa* . Mycologia 97: 872–879. 16457356 10.3852/mycologia.97.4.872

[43] Gil-ad NL, Bar-Nun N, Mayer AM (2002) The possible function of the glucan sheath of *Botrytis cinerea*: effects on the distribution of enzyme activities. FEMS Microbiol Lett 199: 109–113. 10.1111/j.1574-6968.2001.tb10659.x 11356576

[44] Doss RP, Potter SW, Soeldner AH, Christian JK, Fukunaga LE (1995) Adhesion of germlings of *Botrytis cinerea* . Appl Environ Microbiol 61: 260–265. 7887606 10.1128/aem.61.1.260-265.1995 PMC167281

[45] Laspidou CS, Rittmann BE (2002) A unified theory for extracellular polymeric substances, soluble microbial products, and active and inert biomass. Water Res 36: 2711–2720. 12146858 10.1016/s0043-1354(01)00413-4

[46] Zheng L, Campbell M, Murphy J, Lam S, Xu JR (2000) The *BMP1* gene is essential for pathogenicity in the gray mold fungus *Botrytis cinerea* . Mol Plant Microbe Interact 13: 724–732. 10875333 10.1094/MPMI.2000.13.7.724

[47] ten Have A, Espino JJ, Dekkers E, Sluyter SCV, Brito N, et al (2010) The *Botrytis cinerea* aspartic proteinase family. Fungal Genet Biol 47: 53–65. 19853057 10.1016/j.fgb.2009.10.008

[48] Espino JJ, Gutiérrez-Sánchez G, Brito N, Shah P, Orlando R, et al (2010) The *Botrytis cinerea* early secretome. Proteomics 10: 3020–3034. 20564262 10.1002/pmic.201000037 PMC3983782

[49] Christiansen MN, Kolarich D, Nevalainen H, Packer NH, Jensen PH (2010) Challenges of determining *O*-glycopeptide heterogeneity: a fungal glucanase model system. Anal Chem 82: 3500–3509. 20387826 10.1021/ac901717n

[50] Halligan BD (2009) ProMoST: a tool for calculating the pI and molecular mass of phosphorylated and modified proteins on two-dimensional gels. Methods Mol Biol 527: 283–98. 19241021 10.1007/978-1-60327-834-8_21 PMC2754287

[51] Frías M, González C, Brito N (2011) BcSpl1, a cerato-platanin family protein, contributes to *Botrytis cinerea* virulence and elicits the hypersensitive response in the host. New Phytol 192: 483–495. 21707620 10.1111/j.1469-8137.2011.03802.x

[52] ten Have A, Mulder W, Visser J, van Kan JA (1998) The endopolygalacturonase gene *Bcpg1* is required for full virulence of *Botrytis cinerea* . Mol Plant Microbe Interact 11: 1009–1016. 9768518 10.1094/MPMI.1998.11.10.1009

[53] van der Vlugt-Bergmans CJB, Wagemakers CAM, Van Kan JAL (1997) Cloning and expression of the cutinase A gene of *Botrytis cinerea* . Mol Plant Microbe Interact 10: 21–29. 9002269 10.1094/MPMI.1997.10.1.21

[54] Olson GM, Fox DS, Wang P, Alspaugh JA, Buchanan KL (2007) Role of protein *O*-mannosyltransferase Pmt4 in the morphogenesis and virulence of *Cryptococcus neoformans* . Eukaryot Cell 6: 222–234. 17142566 10.1128/EC.00182-06 PMC1797945

[55] Büttner P, Koch F, Voigt K, Quidde T, Risch S, et al (1994) Variations in ploidy among isolates of *Botrytis cinerea*: implications for genetic and molecular analyses. Curr Genet 25: 445–450. 8082191 10.1007/BF00351784

[56] Delcan J, Moyano C, Raposo R, Melgarejo P (2002) Storage of *Botrytis cinerea* using different methods. J Plant Pathol 84: 3–9.

[57] Benito EP, ten Have A, van’t Klooster JW, Van Kan JAL (1998) Fungal and plant gene expression during synchronized infection of tomato leaves by *Botrytis cinerea* . Eur J Plant Pathol 104: 207–220.

[58] Larkin MA, Blackshields G, Brown NP, Chenna R, McGettigan PA, et al (2007) Clustal W and Clustal X version 2.0. Bioinformatics 23: 2947–2948. 17846036 10.1093/bioinformatics/btm404

[59] Applied Biosystems (2008) Guide to performing relative quantitation of gene expression using real-time quantitative PCR. Available: http://tools.invitrogen.com/content/sfs/manuals/cms_042380.pdf. Accessed 6 March 2013.

[60] Schmittgen TD, Livak KJ (2008) Analyzing real-time PCR data by the comparative C(T) method. Nat Protoc 3: 1101–1108. 18546601 10.1038/nprot.2008.73

[61] Sambrook J, Russell DW (2001) Molecular Cloning. A laboratory manual. Cold Spring Harbor, New York: Cold Spring Harbor Laboratory Press. 2344 p.

[62] González C, Noda J, Espino JJ, Brito N (2008) Drill-assisted genomic DNA extraction from *Botrytis cinerea* . Biotechnol Lett 30: 1989–1992. 18594767 10.1007/s10529-008-9790-6

[63] Shevchuk NA, Bryksin AV, Nusinovich YA, Cabello FC, Sutherland M, et al (2004) Construction of long DNA molecules using long PCR-based fusion of several fragments simultaneously. Nucleic Acids Res 32: e19. 14739232 10.1093/nar/gnh014 PMC373371

[64] Yu JH, Hamari Z, Han KH, Seo JA, Reyes-Domínguez Y, et al (2004) Double-joint PCR: a PCR-based molecular tool for gene manipulations in filamentous fungi. Fungal Genet Biol 41: 973–981. 15465386 10.1016/j.fgb.2004.08.001

[65] Van Kan JAL, van’t Klooster JW, Wagemakers CAM, Dees DCT, van der Vlugt-Bergmans CJB (1997) Cutinase A of *Botrytis cinerea* is expressed, but not essential, during penetration of gerbera and tomato. Mol Plant Microbe Interact 10: 30–38. 9002270 10.1094/MPMI.1997.10.1.30

[66] Hamada W, Reignault P, Bompeix G, Boccara M (1994) Transformation of *Botrytis cinerea* with the hygromycin B resistance gene, *hph* . Curr Genet 26: 251–255. 7859308 10.1007/BF00309556

[67] Wessel D, Flügge UI (1984) A method for the quantitative recovery of protein in dilute solution in the presence of detergents and lipids. Anal Biochem 138: 141–143. 6731838 10.1016/0003-2697(84)90782-6

[68] Neuhoff V, Arold N, Taube D, Ehrhardt W (1988) Improved staining of proteins in polyacrylamide gels including isoelectric focusing gels with clear background at nanogram sensitivity using Coomassie Brilliant Blue G-250 and R-250. Electrophoresis 9: 255–262. 2466658 10.1002/elps.1150090603

[69] Gravel P, Golaz O (1996) Identification of glycoproteins on nitrocellulose membranes using lectin blotting. In: Walker JM, editor. The protein protocols handbook. New York: Humana Press. 603–617.

[70] Varela C, Mauriaca C, Paradela A, Albar JP, Jerez CA, et al (2010) New structural and functional defects in polyphosphate deficient bacteria: A cellular and proteomic study. BMC Microbiol 10: 7. 20067623 10.1186/1471-2180-10-7 PMC2817675

