# Supplementary figures and images for: Botrytis cinerea Protein O-Mannosyltransferases Play Critical Roles in Morphogenesis, Growth, and Virulence
Source: PLoS One. 2013 Jun 6;8(6):e65924. doi: 10.1371/journal.pone.0065924 (PMC3675079; doi:10.1371/journal.pone.0065924)

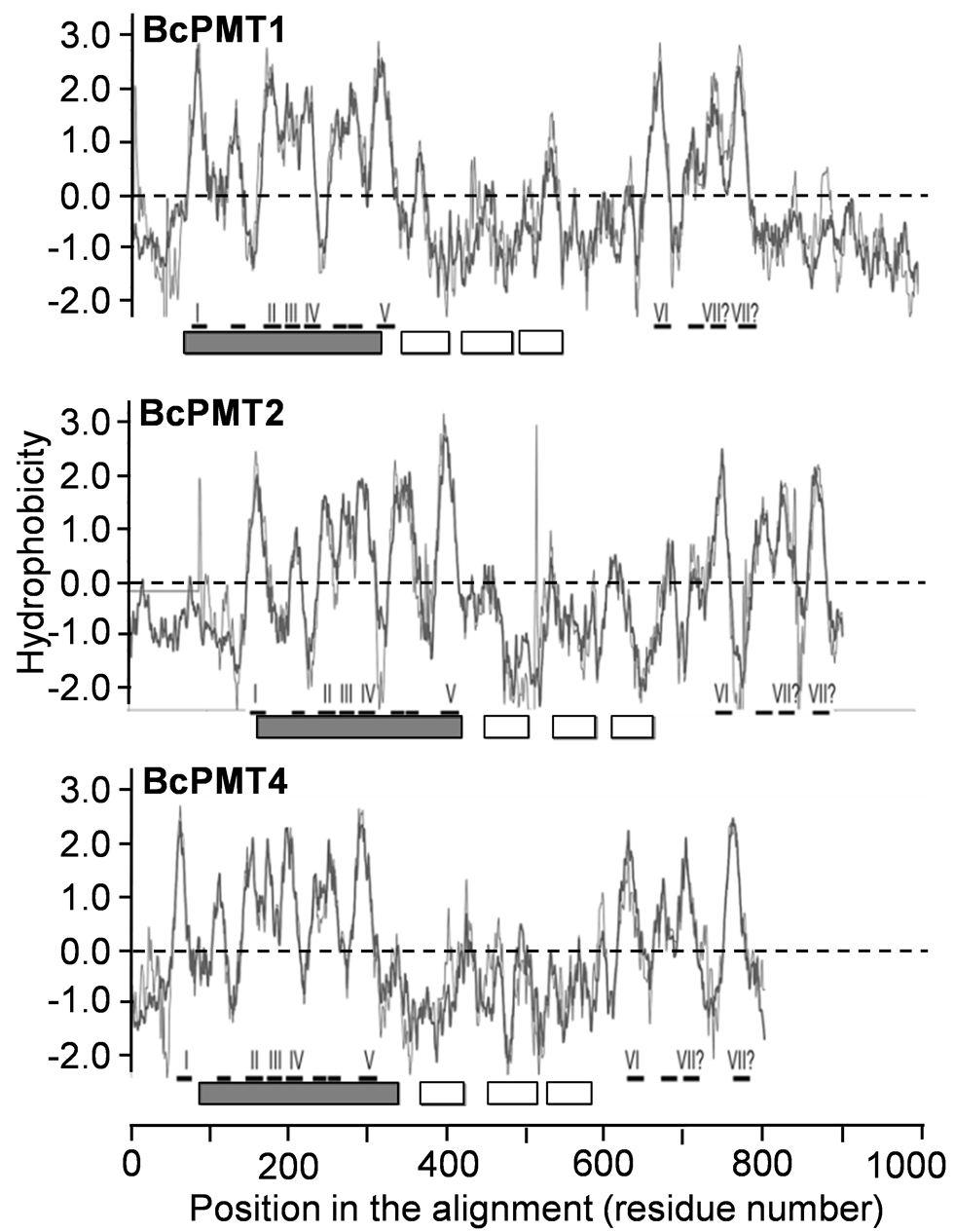

Supplement: Figure S1 — Putative topology of B. cinerea PMTs. The hydrophobicity of each BcPMT (thick line), calculated with the Kyte-Doolittle scale and a window of 15 residues, is plotted alongside the average hydrophobicity of the corresponding PMT subfamily (thin line) derived from the alignment (Figure S2). Black lines below plot indicate the transmembrane regions predicted for the alignment, and those corresponding to the 7 transmembrane domains of S. cerevisiae PMTs are marked with roman numerals (question marks indicate that it is not clear which one of the two regions marked as VII is the last transmembrane domain in S. cerevisiae [14]). PMT domains (grey boxes) and MIR subdomains (white boxes) detected by InterProScan are also marked. (TIF) [file pone.0065924.s001.tif]

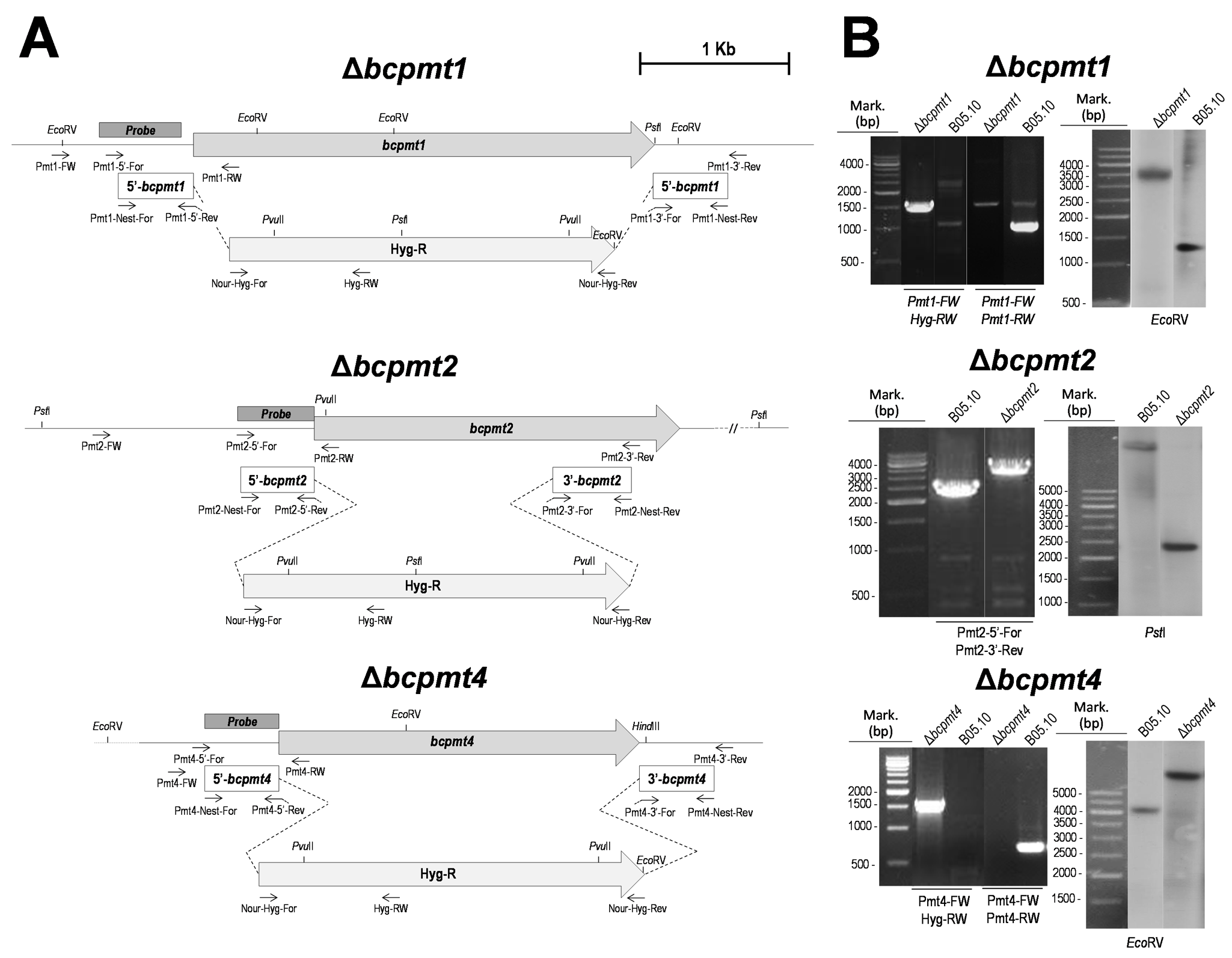

Supplement: Figure S3 — Generation of the three bcpmt knockout mutants. A) Strategy used to generate the three Δbcpmts knock-out mutants. The DNA constructs used in the transformation, shown below each bcpmt gene, contain the hygromycin resistance cassette (Hyg-R) flanked by two regions of the target gene. The position of all primers used to generate the constructs and to check the transformants are indicated (arrows). B) PCR and Southern-blot analysis carried out to check the mutants. Primers used in the PCRs (left panels) are indicated below each picture. Southern-blots were carried out with the indicated genomic DNAs and restriction enzymes, and with the probes indicated in A. (TIF) [file pone.0065924.s003.tif]

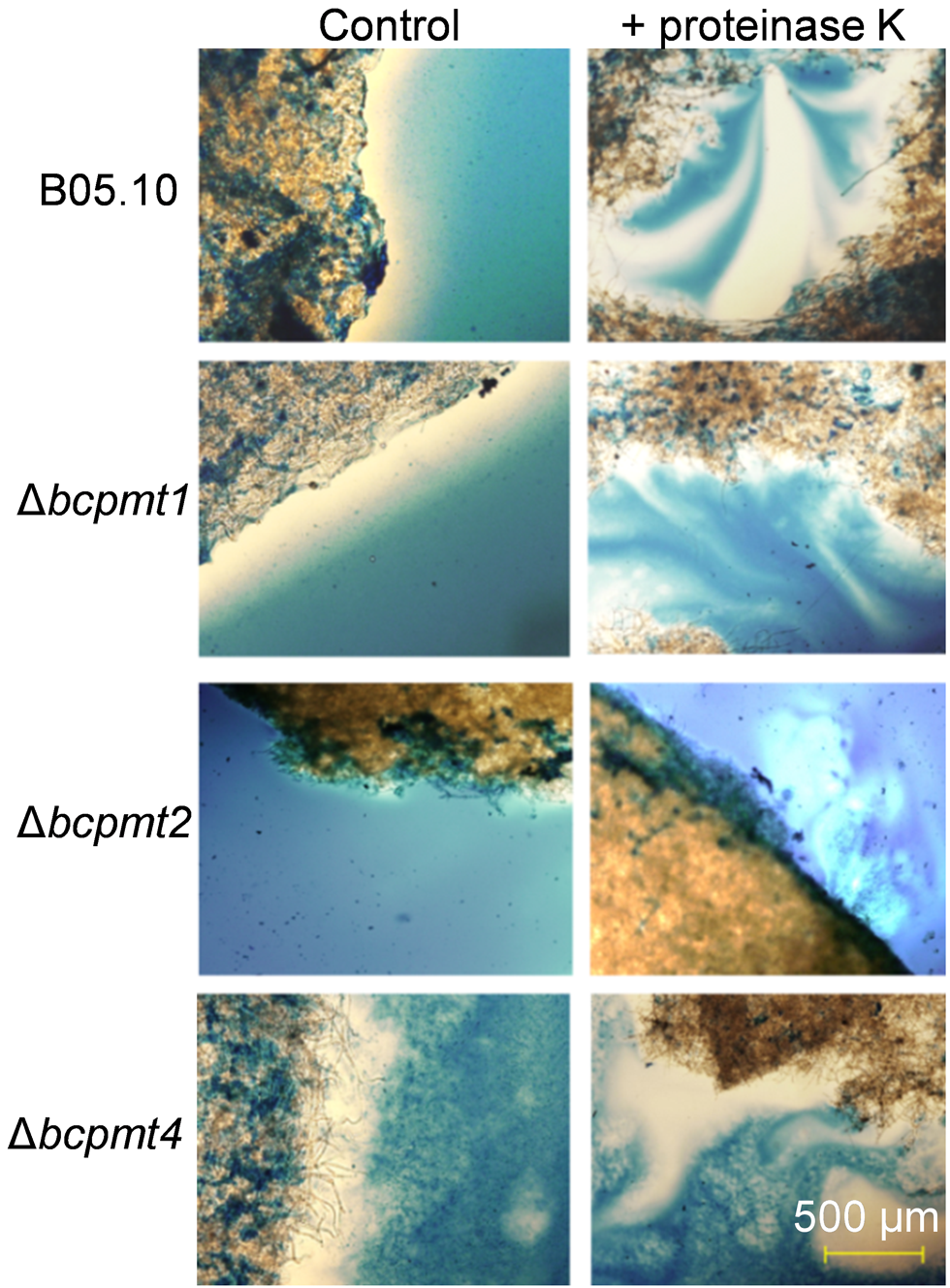

Supplement: Figure S4 — Partial degradation of the extracellular matrix by treatment with proteinase K. Mycelia were grown in liquid YGG medium without shaking, transferred to 0.5X phosphate buffer saline and incubated with 0.4 mg ml−1 proteinase K for 4 hours before staining with India ink. Controls received no proteinase K. (TIF) [file pone.0065924.s004.tif]
