# Supplementary material for: Botrytis cinerea Protein O-Mannosyltransferases Play Critical Roles in Morphogenesis, Growth, and Virulence
Source: PLoS One. 2013 Jun 6;8(6):e65924. doi: 10.1371/journal.pone.0065924 (PMC3675079; doi:10.1371/journal.pone.0065924)
Supplement: Figure S2 — Alignment of fungal PMTs. Proteins were aligned by PRALINE, making use of the transmembrane regions predicted by PHOBIUS (on green background). The regions homologous to the 7 experimentally-determined transmembrane domains of S. cerevisiae Pmt1p are marked with roman numerals. Question marks indicate that it is not clear which region is the last transmembrane domain of Pmt1p. The catalytically-important ER-luminal loop 1 (red boxes), loop 5 (blue boxes), and DE-motif (yellow boxes) are also shown in the alignments. First two letters in protein names indicate the fungus it comes from (Sc: Saccharomyces cerevisiae; Ca: Candida albicans; Sp: Schizosaccharomyces pombe; Um: Ustilago maydis; Cn: Cryptococcus neoformans; Bc: Botrytis cinerea; An: Aspergillus nidulans; Af: Aspergillus fumigatus). Accession numbers can be found in Table S1. (PDF) [file pone.0065924.s002.pdf]

ALIGNMENT OF PMT1 SUBFAMILY MEMBERS

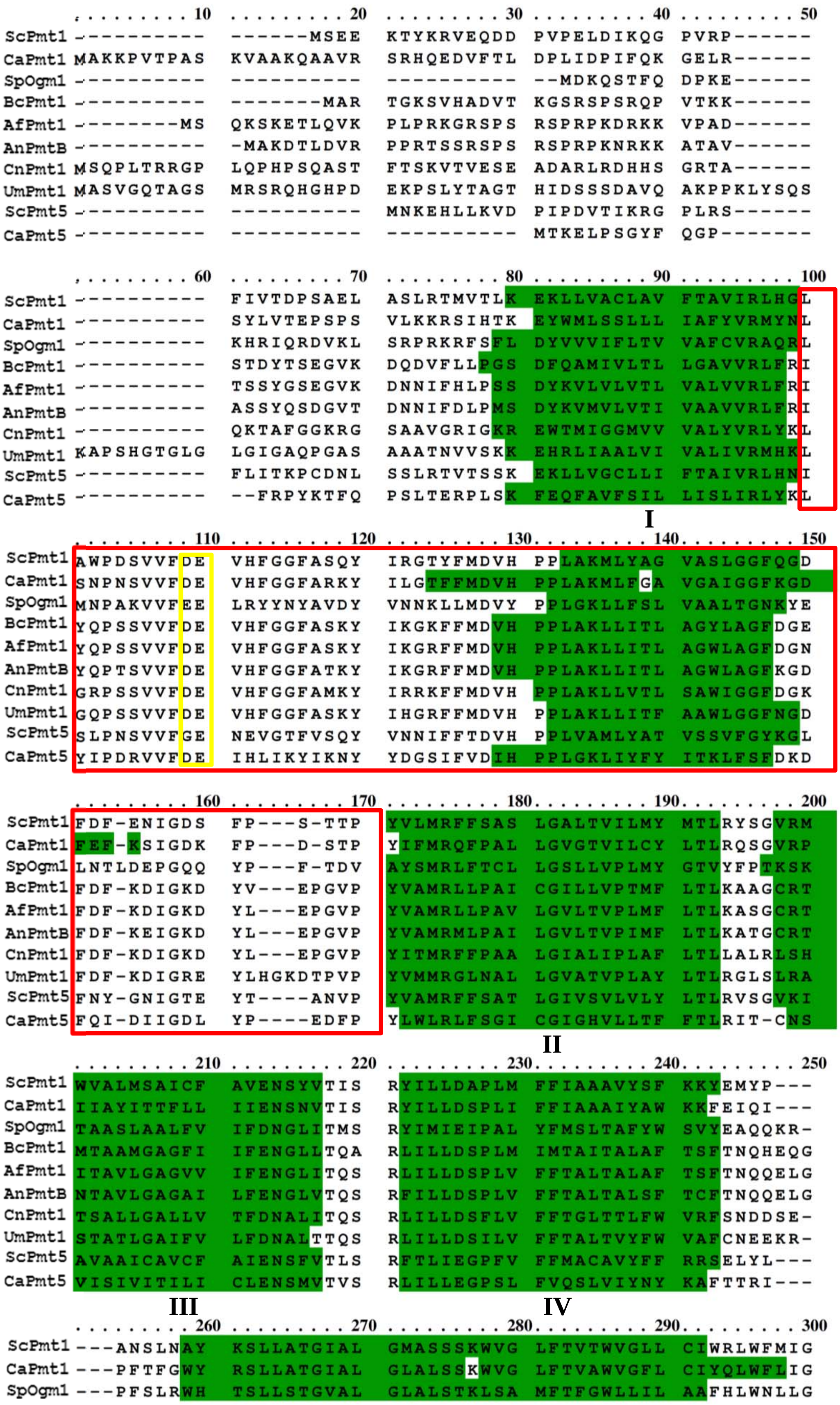

```
BcPmt1 PTKAFGLSWW FWLVMTGFGL GATASVKWVG LFTIAWVGSL TVLQLWVLLG
AfPmt1 PSHAFRGPWW FWLAATGLSL GATLSVKWVG LFTVAWVGSL TVLQLWVLWG
AnPmtB PSHAFRGPWW FWLVATGFSL GATLSVKWVG LFTVAWVGSL TILQLWVVLG
CnPmt1 -GRAFTKPWW INLLCTGLSL GAVVSCKWVG LFTIAMIGVG TLRQLWLLLG
UmPmt1 --SPFSTKWW ALLSLTGLSL GAVASSKW-- ---------- ----LWEHLG
ScPmt5 ---PNSCKAN KSLLAASIAL GFAVSSKWAG LFTIAWAGII VLWRVWFMIG
CaPmt5 ---PFTGCWY FNLFVTGIAL GLNISLKISG LFTFAWVGIL TCVQLWEILG

       .........310.......320.........330.........340.........350
ScPmt1 DLT-KSSKSI FKVAFAKLAF LLGVPFALYL VFFYIHFQSL TLDGDGASFF
CaPmt1 DLS-VSTKKI WGHFFARGII LLGVPIALYL GFFAIHFQLL NKEGDGGAFM
SpOgm1 DLS-VPMYRI VKHLFSYIFY LIGVPITVYL AVFAVHSHIA YKASVADAFL
BcPmt1 DAKTVTPRIF AKHFVARIFC LIIIPLTFYM AMFGIHFLCL VNPGDGDGFM
AfPmt1 DAQNVTPRLW FKHFFARVFC LIIVPLGIYC GMFAIHFLCL VNPGDGDGFM
AnPmtB DTNNVTPRLW FKHFFARVFC LIVIPLAFYC SMFAIHFRCL VNPGEGDGFM
CnPmt1 NLK-VTPRQY IRHFAARTLC LIVVPLTFYM LMFRIHFWIL NESGDGDGFM
UmPmt1 DVR-QPVRVV ARHFCARAIC LISLPMLVYL VSFGIHLGLL WRSGEGDPFM
ScPmt5 DLS-RPIGSS IKYMAFQFTC LLAIPAFIYF LIFSVHIKTL NVNGISSSFF
CaPmt5 DLR-ISIWQF IKHLVLRVVA FIMVPLTIYC SVFYIHFENL PNEGPGSGFL
                            V
       .........360.........370.........380.........390.........400
ScPmt1 SPEFRSTLKN NKIPQNVVAD VGIGSIISLR HLSTMGGYLH SHSHNYPAGS
CaPmt1 SSAFRAGLQG NKIPRDITEQ VGLGSVVTIR HVDTQGGYLH SHEHFYQTGS
SpOgm1 PPEHRHALAG NRF-DDQFAD VAYGSLVTIR NAIPEHGYLH SSELLYPEGT
BcPmt1 SSEFQATLNS KSM-QDVPVD VAFGSRVSIR HHNTQGGYLH SHNLMYPTGS
AfPmt1 SSEFQATLNS KGM-QDVPAD VVFGSRVSIR HLNTQGGYLH SHAHMYPTGS
AnPmtB SSEFQATLNS KGM-AAVPAD VVFGSRLSIR HHNTQGGYLH SHSHMYPTGS
CnPmt1 SSEFQHTLQG HGM-ADTFAD VGFGSKVSIR HVNTQGGYLH SHPHPYPGGS
UmPmt1 SSAFQHTLHG HQM-QDTYAD VALGSTVSIK HLNTQGGYLH SHVATYPAGS
ScPmt5 PAEFRKTLKY NNVIKETVAE VAVGSAVSLN HVGTAGGYLH SHLHNYPAGS
CaPmt5 TPHFRSTLDD YQQ---QPLQ VLYGSTITIK HNAL-EKYLH SHDLTYPRGS

       .........410.........420.........430.........440.........450
ScPmt1 EQQQSTLYPH MDANNDWLLE LYNAPGES-- ---------- ---------L
CaPmt1 KQQQITLYPH LDSNNKWLIE PYNG-TIH-- ---------- ---------N
SpOgm1 EQQIISLVDE PNQNALWIIE HEHSQDNN-- ---------- ---------R
BcPmt1 KQQQITLYPH KDENNIWLLE NQTQPLDING QPIN------ --GSLAWDAL
AfPmt1 KQQQITLYPH KDENNVFILE NQTQPLGPFG QVE------- ---GPYAWDN
AnPmtB KQQQITLYPH KDDNNLFIAE NQTQPLDANG AEIP------ ---GPFAWDN
CnPmt1 KQQQITLYPH RDDNNVWRIV NASAPDGPAS Y--------- ------PWDE
UmPmt1 QQQQITLYPH SDDNNDWLIV KAPGPEDAAP KLDDKGHPLR PEDEVSRWHQ
ScPmt5 MQQQVTLYPH IDQNNKWIIE LAEHPNEN-- ---------- ---------V
CaPmt5 NLQQVTLYDF PDVNNEWVIE TKQKYNEEK- ---------- --------LM

       .........460.........470.........480.........490.........500
ScPmt1 TTFQNLTDGT KVRLFHTVTR CRLHSHDHKP PVSESSDWQK EVSCYGYSGF
CaPmt1 ETFVPLINGM KIRLKHINTG RRLHSHDEKP PVSERD-WQK ECSCYGYDGF
SpOgm1 SNIELLKDGS VVRLRHVMTG RALHSHEHKP IVSNND-WQL EASAYGGFGF
BcPmt1 PETKLIQDGD VLKLYHQPTH RRLHSHDVRP PVTEAD-WQN EVSAYGYEGF
AfPmt1 ITTEYIQDGA VVRLYHAMTH RRLHSHDERP PVTDVD-WQF EVSAYGYEGF
AnPmtB LTTNYIEDGA VIRLHHLMTH RRVHSHNERP PVTDVD-WQF EVSAYGYEGF
CnPmt1 LPFEYVLTGT KIRLEHVTTE KRLHSHDIRP PVSEVD-FQN EVSGYGFPGF
UmPmt1 PPIRYLTHGM EIRLIHKTTD KRLHSHDTNR PPVTESDYQN EVTAYGFEGF
ScPmt5 TSFQNLTDGT IIKLRQLKNG CRLHSHDHKP PVSQNADWQK EVSCYGYEGF
CaPmt5 TDQREVKDGD VVRLYHKATG HYLHVNDIRP PISEHE-YSY EVNGNETRGL

       .........510.........520.........530.........540.........550
ScPmt1 DGDANDDWVV EIDKKNS--A PGVAQERVIA LDTKFRLRHA MTGCYLFSHE
CaPmt1 AGDANDDWVV EIVNYRS--Q KGEAQTFVKA INTIFRLRHA MTGHYLFSSE
SpOgm1 EGDANDLFRI QILEKKS--K HATSNGTVET LNTKFRLIHV FANCELMSSH
BcPmt1 EGDANDFFRV EIIKKMS--D GELAKTRLRT IQTKFRLVHI MTGCVLFSHK
AfPmt1 PGDANDLFRV EIVKSKS--D GEEAKKRLRT IESKFRLVHV MTGCVLFSHK
AnPmtB PGDANDYWRV EIVKSLS--D GEEAKKRLRT IQSKFRLVHV MTGCVLFSHK
CnPmt1 AGDANDDFIV EITKRTRGKN DKQAKHRLKT LRSEFRLRHA LSGCYLFSHK
UmPmt1 GGDANDNFHV EIVAGDK--S DPYSSTRVRA LRTHFRLRHT LTGCYLFSHK
ScPmt5 EGDINDDWII EIDKKRS--E PGPAQEHIRA IETKFRLKHY LTGCYLFSHP
CaPmt5 LGNEDYEFKI RMLVKKPHAE NDLPLIKLRT TETIFQLIHQ ATRCNLMSHE

       .........560.........570.........580.........590.........600
ScPmt1 VKLPAWGFEQ QEVTCASSGR HDLTLWYVEN NSNPLLPEDT K-RI-SYKPA
CaPmt1 VKLPEWGFGQ QEVTSASQGK RALTHWYIET NENSILPPSE A-KIINYPKL
SpOgm1 RRFPDWGDYQ REVTCCRNCV ERSTTWFIES NYHDGLPSDS R-KI-TYRKP
BcPmt1 VKLPEWASEQ QEVTCAKGGT LPNSIWYVEQ NEHPQLGADA E-KV-NYRNP
AfPmt1 VKLPEWGFDQ QEVTCARGGT LPNSLWYIES NHHPMLPEDA E-KV-NYRNP
AnPmtB VKLPDWGFDQ QEVTCAKGAS LPNSIWYIES NKHPMLPPDA E-KV-TYRNP
```

```
CnPmt1 VKLPDWGYEQ  QEVTCNKNPT  WENSLWYIET  NQHVQLPIDA  E-RV-NYEKP
UmPmt1 VTLPDWGFGQ  QEVTCNKNPT  MPNSLWYVET  NTHPLISPET  QPDLVNYRRP
ScPmt5 EKLPEWGFGQ  QEVTCAYFAR  EDLTSWYIEE  NENEISLPNP  --EKVSYKKM
CaPmt5 QKLPDWGEYQ  NEVLCVKEPT  IPNTLWYVES  SSHPLLKDTK  --KLKTFPKF

       .........610 .........620 .........630 .........640 .........650
ScPmt1 SFISKFIESH  KKMWHINKNL  VEPHVYESQP  TSWPFLLRGI  SYW-------
CaPmt1 SLWQKVVESH  KRMWKINQGL  TSHHHWQSSP  SEWPLLLRGI  NYW-------
SpOgm1 GFLESFVEHN  KLMWLKDRKM  GDGHVYESSA  LTWPLLLGPL  RFF-------
BcPmt1 GFFGKFWELN  KVMWHTNAGL  VESHAWDSRP  DSWPILKRGI  NFW-------
AfPmt1 GFFGKFWELQ  KVMWTTNAGL  TESHAWDSRP  PSWPTLLRGI  NFW-------
AnPmtB GFFGKFFELQ  KVMWTTNAGL  TDSHAWDSRP  PSWPTLLRGI  NFW-------
CnPmt1 SFFEKFFELN  AVMWRTNAGL  TERHAYDSRP  QHWPWLRRGI  NFW-------
UmPmt1 SFLERFWELQ  SVMWETNAGL  TDRHAYDSRP  GTWPMLKRGI  NFW-------
ScPmt5 SFWQKFVAIH  KFMFYLNNYM  DTSHAYSSEP  KTWPLMLRGI  DFW-------
CaPmt5 SFWSKLIETH  KVMFNLNKGF  TNPHPYASKP  LDWPLLSRGI  AFFSNYNLKS

       .........660 .........670 .........680 .........690 .........700
ScPmt1 -GENNRNVYL  LGNAIVWWAV  TAFIGIFGLI  VITELFSWQL  GK----PILK
CaPmt1 -NKEHKQVYL  LGNAVTWWAA  TLSIITFGTY  VLVTVFRWHL  GT----PLST
SpOgm1 -YEQHLQVFF  MGNPFVWYSV  ISLVAFFVIV  QIFCLARWNL  GY----NDFG
BcPmt1 -GKDNRQIYL  IGNPVIWWSS  TLAVVIFVAF  KGLAVLRWQR  GF----RDYD
AfPmt1 -GRDHRQIYL  LGNPFIWWSS  TAAIVVYIVF  KGIAVIRWQR  SC----GDYR
AnPmtB -GRDHRQVYL  FGNPFVWYSS  TLAVLVYVIF  KGISLLRWQR  NC----GDYR
CnPmt1 -VKDHRQVYL  IGNPVVWWSS  TAAIVAYLAV  RGFLVLRAQR  GY----RDLH
UmPmt1 -TKDHRQIYL  IGNPIVWWSA  FGSVLAYLGA  RGVLMLRAQR  GY----NDLK
ScPmt5 -NENGREVYF  LGNAVLWWSV  TAFICTFIIG  VAVELLAWKL  GV----NILR
CaPmt5 IDEESSLIYY  LGNVAIYYSV  FFVGLIAIFK  CAIYSFIKLN  PYASPPSSSK
                                 VI
       .........710 .........720 .........730 .........740 .........750
ScPmt1 DSKVVNFHVQ  VIHYLLGFAV  HYAPSFLMQR  QMFLHHYLPA  YYFGILALGH
CaPmt1 NKHVFNFNVQ  TFSYVLGWAL  HYLPFFIMGR  QLFLHHYLPA  LYFGILALGH
SpOgm1 PSAF-HYNYN  IGKFVVAWLL  HWAPYILETD  RVFLYHYLPA  LYFGIAALGV
BcPmt1 NPVFKRFDYE  IGTSVLGWAF  HYFPFYLMQR  QLFLHHYFPA  LYFAIIALCQ
AfPmt1 NVNFKRFDYE  VGTSVLGWAF  HYFPFYLMAR  QLFLHHYFPA  LYFALMALCQ
AnPmtB NPAFKRFDYE  IGTSVLGWGF  HYFPFYLMAR  QLFLHHYFPA  LYFAILALAQ
CnPmt1 QPKLAFYDDI  CAFCVIGWAL  HYFPFYLMQR  QLFLHHYLPA  LYFAILLFCT
UmPmt1 DSRVRFYDQT  CGFLVLGWAL  HYLPFFLMNR  QLFLHHYLPA  LYFSILLLAV
ScPmt5 DKHIINFHYQ  VFQYLLGFAA  HYFPYFFVGQ  KLFLYDYLPA  YYFGILAFGH
CaPmt5 SSPYANFYNN  SWPYLVGWFI  NYIPYCLMSR  NLYLHHYLSA  LNFGILLLSQ
                                                         VII?
       .........760 .........770 .........780 .........790 .........800
ScPmt1 ALDIIVSYVF  ----RSKRQM  GYAVVITFLA  ASVYFFKSFS  PIIYGTPWTQ
CaPmt1 FFEIFTGYLT  -SRSKYFQQV  AFVLVGLFSI  LSLVFYVNYS  SLIYGTPWTK
SpOgm1 SWSFLGNAV-  ----FGNRTA  YKALSVIIMA  LMFLVYRLYS  PFTYMTTLTK
BcPmt1 IFDFITARVP  GIGLRERPFI  GRIGAIIFLT  LSMVVFALYS  PLAYGNPWTQ
AfPmt1 EFDFIANRFK  SFGLSSRPII  GKGLVAVFLA  LSIFTFTLYS  PLVYGNPWTQ
AnPmtB EFDFIT----  ----------  ---NRIRSLG  LTIFAFTVYS  PLIYGNPWTR
CnPmt1 VFDYATSAL-  ------KPKI  RTNIAIVILI  LALWSWNHWS  SLAYAGEWTK
UmPmt1 VFDFATSTL-  ------RRRF  RLVAGLLATL  VVLAGFVRYS  ALTYGSDWTL
ScPmt5 ALDLISTYIS  ----NKRNNT  GYIVVAIFMV  VCFYFFSEHS  PLIYATGWSS
CaPmt5 YLNYRVAK--  ------NKII  GGIITATIFV  SAIYCFYEFI  PITYGLPWTL
                                         VII?
       .........810 .........820 .........830 .........840 .........850
ScPmt1 ELCQKSQWLS  GWDYNCNTYF  SSLEEYKNQT  --LT------  -----KRESQ
CaPmt1 ASCELTKPFS  GWDYNCGTFF  DTLGEYDIQE  KSLA------  -----SESEI
SpOgm1 SSCRALELKG  SWNFHCNTYL  DNLSDYKFSS  DAGET----Y  FEKAAPHPFV
BcPmt1 SACKQVKIFE  KWDWDCNTFL  NNYSDYATQY  VNANADVHKT  DSPLIPTNAA
AfPmt1 DACKKVKLVS  TWDFDCNTFY  TDLGQYVTQF  LNAN------  PIASSTPSAQ
AnPmtB DACKQVKLLK  SWDFDCNTFY  TDLNQYVTHF  SSV-------  ------NSAV
CnPmt1 GACENGKWLR  TWDFSCNDFY  ENTSMYNSQS  AISSEKIAPT  DLGPDSATTT
UmPmt1 AACEKARWRK  AWDFNCAEFP  RDLVEFQNYP  PSVQ------  -----TLDWA
ScPmt5 NLCKRSKWLG  SWDFYCNSLL  LSDSHYELNA  ES--------  ----------
CaPmt5 DQCNSHKWFP  NWDIDCMTYT  G---------  ----------  ----------

       .........860 .........870 .........880 .........890 .........900
ScPmt1 PAATSTVEEI  TIEGDGPSYE  DLMNEDGKKI  FKDTEGNELD  PEVVKKMLEE
CaPmt1 PTETVVVEAK  QTPKAEPKLA  KQDDHIESPA  AAEPVEEKEV  KEEVEQLAPP
SpOgm1 YSEDTAKKSE  GDTPLNKNLN  DYYPSWDQRV  EAGYKLAAQQ  KAEQEAREAA
BcPmt1 EIPAQAGAGE  QKPLVEQESV  SGAPEGKPEG  LVSP-PPAAP  VASHSIVSRE
AfPmt1 VNPVPELPVQ  NSPVKEPPVA  PPQQHHQEVH  KEAQ-EASVT  SQAQQPRGTM
AnPmtB PTTAASIPQS  PAPVKAEPKQ  EQKEQKEAKV  ESQE-PSITS  SSIPKIRGTQ
CnPmt1 LIEEVPEPIQ  NVFQQDPPPE  EKTVAPVGPA  PEVQMEESSA  VFIPKGEEAP
UmPmt1 INKNKAHPVA  ALALNSTHDA  GGVAAEPGRH  AFDQVPIRAM  QSSAAEAIQH
ScPmt5 ----------  ----------  ----------  ----------  ----------
```

```
CaPmt5 ----------  ----------  ----------  ----------  ----------

       .........910.........920.........930.........940.........950
ScPmt1 EGANI-----  ----------  ----------  LKVEKRAVLE  ----------
CaPmt1 LAVDFEEETP  KVEDPQVADV  ---DASSNDE  KSVEEKQQQE  QQQEQEQV--
SpOgm1 EKAASEAAER  SSSEAAASSS  SESVAAASVE  AERLAMEADE  FNGASETVD-
BcPmt1 EKVEYRDQDG  NILDPEAVKS  L--EGKVSFK  TRYETRTRMV  DAQGNEINPP
AfPmt1 ARVEYRDQHG  NVLDEALVAS  LAREGKVSFE  TRHETRTRLE  HAHEVEMID-
AnPmtB ARVEYRDQQG  NILDEAVVDS  LRKEGKISIE  TRHETRTRLE  HGHLVDVVD-
CnPmt1 LAEDLRAPVG  NDAGDPAVT-  ---DGEDDGG  WHGGAEEGQH  RQEKDEVKI-
UmPmt1 LKNGLQHDLD  YVKDKVGLGS  VHAPNVDPAG  LDQQQIEHVM  LQGTAEV---
ScPmt5 ----------  ----------  ----------  ----------  ----------
CaPmt5 ----------  ----------  ----------  ----------  ----------

       .........960.........970.........980.........990.........1000
ScPmt1 ----------  ----------  ----------  ----------  ----------
CaPmt1 ------EDES  VHQVQQ----  ----------  ----------  ----------
SpOgm1 ---GASVEAE  RSAMEAAALN  NAAE------  ----STEVVG  SSPESVASEQ
BcPmt1 EEDAVGVAPP  HPDVEGSDPQ  TVGKAEPDVQ  EKPVAQRNVQ  ADESKEQSIE
AfPmt1 ----GRIAPP  HPDVEGQNPE  TVKD-----Q  EHQAAGDSPA  SAAVGERSVK
AnPmtB ----GKVAPP  HPDVEGQNPE  TQDK------  PEEIVDDSPA  SAADAGSSVG
CnPmt1 ---KEGKAPV  GAKVDIPDMG  LDEE------  QKLLVDQIMD  EQE-------
UmPmt1 ------VAAS  SATLEAPPTE  ----------  ---------P  SQEAKFDQHA
ScPmt5 ----------  ----------  ----------  ----------  ----------
CaPmt5 ----------  ----------  ----------  ----------  ----------

       .........1010.........1020..
ScPmt1 ----------  ----------  --
CaPmt1 ----------  ----------  --
SpOgm1 EENVAESAQA  RVE-------  --
BcPmt1 KSDDGAAKPA  SEGNEATAA-  --
AfPmt1 EPSSPEPKPA  SEAKEATQN-  --
AnPmtB EPNSPEPKPA  SEGNEATR--  --
CnPmt1 ----------  ----------  --
UmPmt1 DQEAHEAADV  VQADRQVILK  DV
ScPmt5 ----------  ----------  --
CaPmt5 ----------  ----------  --
```

ALIGNMENT OF PMT2 SUBFAMILY MEMBERS

```
        .........10 .........20 .........30 .........40 .........50
ScPmt2 ----------- ----------- ----------- ----------- -----------
CaPmt2 ----------- ----------- ----------- ----------- -----------
ScPmt3 ----------- ----------- ----------- ----------- -----------
ScPmt6 ----------- ----------- ----------- ----------- -----------
UmPmt2 MLPTSRADAP  KRSSYTNITI  DHPHAVSSAT  SPYATTAALD  SDQDLPAPYR
CnPmt2 ----------- ----------- ----------- ---------MH  RGIPSP-PAT
CaPmt2 ----------- ----------- -MATGYSTGV  SPFDLDENNH  ND---------
SpOgm2 ----------- ----------- ----------- ----------- -----------
BcPmt2 ----------- ----------- ----------- ----------- -----------
AnPmtA ----------- ----------- ----------- ----------- -----------
AfPmt2 ----------- ----------- ----------- ----------- -----------

        .........60 .........70 .........80 .........90 .........100
ScPmt2 ----------- ----------- ----------- ----------- --------MSS
CaPmt2 ----------- ----------- ----------- ----------- --------MST
ScPmt3 ----------- ----------- ----------- ----------- -----------
ScPmt6 ----------- ----------- ----------- ----------- -------MSKA
UmPmt2 KRYIDSHDTL  LPTHSPPSST  YLSQDSPGRM  SSPAANSRRF  AAGFDPYSPA
CnPmt2 RRHTAIQHTP  WENRVPPSAY  GSARPHSAFL  EATPHDDAEL  PLFKDTNMEL
CaPmt2 ----------- ----------- ----------- ----------S  IHHRHQNHHS
SpOgm2 ----------- ----------- ----------- ----------- -----------
BcPmt2 ----------- ----------- ----------- ----------- -----------
AnPmtA ----------- ----------- ----------- ----------- -----------
AfPmt2 ----------- ----------- ----------- -----MSAPIC  GVGMFTPERK

        .........110.........120.........130.........140.........150
ScPmt2 SSSTGYSKNN  AAHIKQENTL  RQRESSSISV  SEELSSADER  DAEDFSKEKP
CaPmt2 SVEPNETEAL  LRKQNDLSTT  ASIEEKYPHQ  QGEAAEDDDD  TLKRTQYDEA
ScPmt3 -----MPYRV  ATGYSEKSTD  DDLIWRTPIV  KEELEDADNF  LKDDAELYDK
ScPmt6 KGTGFSSIDT  EDENLRERYV  NQPKANASDI  QDEQLDCFEQ  LEEKHRTKKN
UmPmt2 TTVDSDHADL  SSDSAALKAK  EAGFQPPSSN  TPKVNKYAHL  IPVHQQRKPP
CnPmt2 KRRFDEKQPV  HLEQPVVDDD  QGKWEKGYGA  GPGIGGRRGL  PPRQRIPGWK
CaPmt2 QSHDSSGERD  DTEIEDIIQK  TSKLNINTST  STKIKNFFFQ  SSNRHDSSNS
SpOgm2 -----MSYEQ  LHAQSGQLRQ  RFPSKHSEIE  DEVANEKEEL  KDATKSALGE
BcPmt2 ---MASDKTA  VASGADHTQT  TRRRNVPSSN  GSLVNTVEVD  DKKTQVKKGK
AnPmtA ---MAEIGFA  STTGASFAPD  VRRRNVRTEG  QARTSGL-IE  PDDKKHKQRP
AfPmt2 LISSSHLIIP  RTRTRTQSRG  LRYGGHTHPV  SPAERHI-LS  PANDPFTEQA

        .........160.........170.........180.........190.........200
ScPmt2 AA-QSSLLRL  ESVVMPVIFT  ALALFTRMYK  IGINNHVVWD  EAHFGKFGSY
CaPmt2 KETAESLKQV  ESILAPIVFT  ALSFFVRFYR  ISVNDHVVWD  EAHFGKFGSY
ScPmt3 VKNESAVSHL  DTIVMPIIFT  VLGMFTRMYK  IGRNNHVVWD  EAHFGKFGSY
ScPmt6 EEYTALK-IL  RDVIGPLLLT  ITSFYLRFQH  IDQNNYVVWD  EAHFGKFGSY
UmPmt2 NFEIVLWTLR  QEEVIGLIYT  LFSLITRLWG  IGNSNVVVWD  EAHFGKFGSH
CnPmt2 GIVQE----H  EEWVWAGVYT  LLSMITRFWR  IGAANYVVWD  EAHFGKFGTH
CaPmt2 PPLREV---F  IKTINPLILT  AISSFVRLYR  IDVANSVVWD  EAHFGKFGSQ
SpOgm2 VKTNKKYYIL  GYFLVPLLLT  VIAGFVRVWK  IADSNVVIWD  EAHFGKFASY
BcPmt2 QSLVGFLDEW  EFLIAPLVFT  IFAFFTRLYK  IGLSPIVTWD  EAHFGKFGSH
AnPmtA NSYLSALADW  EPLIAPILLT  VLSMFTRMYR  IGRSNIVTWD  EAHFGKFGSH
AfPmt2 NSFYTILDSW  EPFIAPIVLT  AFAVFTRMYR  IGRSNIVTWD  EAHFGKFGSH
                        I

        .........210.........220.........230.........240.........250
ScPmt2 YLRHEFYHDV  HPPLGKMLVG  LSGYLAGYNG  SWDFPSGEIY  PDYLDYVKMR
CaPmt2 YLRHEFYHDV  HPPLGKMLVG  LSGYLAGYNG  SWDFPSGEKY  PDYIDYTKMR
ScPmt3 YLRHEFYHDV  HPPLGKMLVG  LSGYLAGYNG  SWDFPSGEVY  PDYIDYVKMR
ScPmt6 YIKHEYYHDV  HPPLGKMLIA  LSEWMAGFDG  QFDFSSNNAY  PENVNFKLMR
UmPmt2 YLQQEFYFDV  HPPLGKMLVG  LAGLISGYRG  QFEFKSGETY  PADVNYIGMR
CnPmt2 YIKRDFYFDV  HPPLGKMLVG  LAGLLSGYQG  NFEFKSGVAY  PEDVPYTAMR
CaPmt2 YLKRQFYFDV  HPPLGKLLIG  LSGYLADYDG  NFDFESSNVY  PDNVNYVFMR
SpOgm2 YLKHEFYFDV  HPPLGKMLNA  VAGKLVGYDG  SFDFSSGATY  PEDLNYKFMR
BcPmt2 YIKREFYFDV  HPPLGKMLVG  LSGYLAGYNG  TFEFKSGEQY  PEDVNYTFMR
AnPmtA YLKREFYFDV  HPPLGKMLVG  LSGLLAGYNG  SFEFKSGETY  PEDLNYTFMR
AfPmt2 YLKREFYFDV  HPPLGKMLVG  LSGLLAGYNG  SFEFKSGEKY  PEDVNFTFMR
```

```
         .........260.........270.........280.........290.........300
ScPmt2 LFNASFSALC VPLAYFTAKA IGFSLPTVWL MTVLVLFENS YSTLGRFILL
CaPmt2 LFNATFSALC VPLAYFTGKE VGFSMFTTWL FTLMVALESS YVTLGKFILL
ScPmt3 LFQAMFSSLC VPLAYFTGRA IGFSRLSVWL FTILVIFENS YATLGKFILL
ScPmt6 QFNATFGALC TPVAFFTAKW MGFNYFTVYL IATMVTLEHS YIVLSKFILL
UmPmt2 VILAMFGVAM VPLAWFTSGG LNWNWRARHL LTLMVLLDNG WLVISRFILL
CnPmt2 VMLASFGVAL VPLAWFTSGE LGWSRWTRHW VTICVLCDIG WLCISRFILL
CaPmt2 IFNCFFGILV TPLAYKTAVI LGYNQFTCWL IAFMVIFEQL SLTLSKFILL
SpOgm2 LWNAAFGTLC IPLVYFTALN FNYSFLAATL CTLMVALDNH LATISRFILL
BcPmt2 AFNAFFGAVT IPLAYYTARE LNFKRPAVWF VTLMVLCENS YTTISRFILL
AnPmtA LFNAAFGVVC VPLAYLTARE LGFRRGTVWL VSLMVLFENS YATISRFILL
AfPmt2 VFNAAFGVAC VPLAYYTARE LGFRKATVWL ISLMVLFENS YATISRFILL
                 II                          III
         .........310.........320.........330.........340.........350
ScPmt2 DSMLLFFTVA SFFSFVMFHN Q--------R SKPFSRKWWK WLLITGISLG
CaPmt2 DSMLLFFTVA TVFCFSRFNN FNN------K SQEFSRKWWK WILLTGVSIG
ScPmt3 DSMLLFFTVS SYFCLAKFHT M--------R KSPFSARWWL WLCLTGLNLG
ScPmt6 DSMLLFFSMT TFACMIKLYT L--------R KQQMTKKWSL WMLLTGLSIG
UmPmt2 DSMLLCFTFT TVHGLVKFLQ Y--------K SAPFTKAWWF WLAFTGASIG
CnPmt2 DSMLLFFTFT TTLGLVKFRN Q--------R HAPFSDDWWI WLVFTGWSIG
CaPmt2 DSMLLFFTVL TMYCLVKVHT LAIARVGSNS KTPLTKLEIK WYILTGISIG
SpOgm2 DSMLLFFIIS TFFCLSRYHV Y--------H KAPFTFYWFK WLFLTGVCIG
BcPmt2 DSMLLCFTFT TVLCWAKFHN L--------Q KQSWTAEWFV WLLLTGVSIG
AnPmtA DSMLLCFTFT TTFCWAKFHR L--------Q HASFSIEWFT WLFLTGVSIG
AfPmt2 DSMLLCFTFM TTMCWAKFHR L--------Q HASFSGQWFT WLFLTGISIG
                 IV
         .........360.........370.........380.........390.........400
ScPmt2 CTISVKMVGL FIITMVGIYT VIDLWTFLAD K--------- SMSWKTYINH
CaPmt2 CTCSVKMVGL FVTTLVGIYT VVDLWNKLSD K--------- SISWTKYIQH
ScPmt3 CAISVKMVGL FIISVVGIYT ISELWNLLSD R--------- SVSWKVYVNH
ScPmt6 CVCSVKWVGL FITVVVGLYT CIELFLLYCD K--------- ELPRIKYYKH
UmPmt2 CVSSVKWVGL FVTALVGVFT VEDLWEKFGD L--------- RMPVRAYVRH
CnPmt2 CVCSVKWVGM FITALVGLYT IEDLWEKFGD L--------- SMPIRTYITH
CaPmt2 CVCSVKWVGL FVTALVGFYT IVDLWIKFYQ TFAIDKKSPK KMSVVNYLIH
SpOgm2 CVCSVKLVGL FITAVVGLYT VDELWCLLND K--------- RVTWKAYAGH
BcPmt2 CVCSVKMVGL FCTTMVGIYT IEDLWNKFGN I--------- RMQKVELASH
AnPmtA CVCSVKWVGL FCTALVGLYT IEDLWNKFGD L--------- KMSEATLAKH
AfPmt2 CVCSVKWVGL FCTALVGLYT IEDLWNKFGD L--------- RMPKTVLVNH

         .........410.........420.........430.........440.........450
ScPmt2 WLARIFGLII VPFCIFLLCF KIHFDLLSHS GTGDANMPSL FQARLVGSDV
CaPmt2 WFARIVALIL VPIFIFMLSF KVHFDLLYKS GTGDANMSSL FQANLAGSDV
ScPmt3 WLARIFGLII IPVCVFLLCF KIHFDLLSNS GPGDSTMPSL FQASLNGTKV
ScPmt6 WLIRIINLIV IPFLIYLYCF KIHFVLLYKS GTGDSTTNTL FQINLEGTQI
UmPmt2 WCARILCLIF LPLTIYMLSF KAHFLILSRS GPGDAQMSSL FQSHLRGNDF
CnPmt2 WVARITCLII LPFIVYASCF KIHFLILNRS GPGDAQMSSL FQAHLRGNDF
CaPmt2 WVVRIFTLII IPMTIYVATF KVHFMVLNHT GPDDGTLSTL LQGSLIGNDL
SpOgm2 WIARVCLLIF LPILIYAFTF WIQFAVLYRS GPGDAQMPSL FQARLEGSPL
BcPmt2 VAIRVICLIV IPIGVYIASF AAHFAILQNS GPGDAQMSSL FQANLRGTEV
AnPmtA FAVRVVGLIL IPALVYIFSF YIHFLILENS GPGDAQMSSL FQANLKGTQV
AfPmt2 LLARVVGLII IPAVVYMLSF YVHFWILENS GPGDAQMSSL FQANLKGTEV
                    V
         .........460.........470.........480.........490.........500
ScPmt2 -GQGPRDIAL GSSVVSIKNQ ALGGSLLHSH IQTYPDGSNQ QQVTCYGYKD
CaPmt2 -GGGPREVSM FHSVITLKNQ GLSGGLLHSH VQTFPEGSKQ QQVTTYGHKD
ScPmt3 -GKGPRDVAL GSSIISIKNQ ALGGALLHSH VQPFPEGSEQ QQVTVYGYSD
ScPmt6 -EAGPRDVAF G-SELTIRSH GLSPNLLHSH IQVYPEGSGQ RQITGYGFAD
UmPmt2 -ALSPPEAAF G-SKITLKNM GYGGGLLHSH VQTYPVGSQQ QQVTCYHYRD
CnPmt2 -AESPLEIAY G-STVTLKNY GYGGGLLHSH VQTLPVGSLQ QQVTCYHYKD
CaPmt2 -QSGPRSVAF G-SLVTIRSQ GLSPNLIHSH PHNYPQGSQE QQVTTYGFKD
SpOgm2 -TKNPIDLMY G-SKFTLKSR NPTGALLHSH VQTYPEGSEQ QQVTGYHHKD
BcPmt2 GKDSPLEIAY G-SRATIKNM GYGGGLLHSH IQTYPEGSNQ QQITCYHHKD
AnPmtA GKDSPLEIAF G-SRVTLKNM GYGGGLLHSH VQTYPEGSSQ QQVTCYHHKD
AfPmt2 GKDSPLEIAV G-SRVTLKNM GYGGGLLHSH VQTYPDGSNQ QQVTCYHHKD

         .........510.........520.........530.........540.........550
ScPmt2 ANNEWFFNRE RGLPSW---- ---------- SENE--TDIE YLKPGTSYRL
CaPmt2 SNNNWIFQRA RGQPYY---- ---------- DTSGNTTDIE YIFDGMHVRL
ScPmt3 ANNEWFFQRI RGVEPW---- ---------- TDAH-NKTIE FVKGGEMYRL
ScPmt6 SNNVWKFEFS RSSGLE---- --------LD QNGTLNGKII PITDGVEVRL
UmPmt2 NNNEFIITPP WNERAL---- --------PA NYSSSTEPVR MLKNNDVIRL
CnPmt2 ENNNWQIVPP WGADPV---- ---------- ---DPDGPIR FLKDGDEIRL
CaPmt2 DNNEFLFEFG VDAGLRNQHA TLENENSTRN GGNDDDYYHV IIHDGDTVRI
SpOgm2 GNNEWMFVPT HGVAYN---- ---------- --YEENDPMN PILNGSVVRL
```

```
BcPmt2 ANNEWWFYPN RSQP-E---- ---------- --FDPEAPLR YVADGDVLRF
AnPmtA ANNDWFIYPN RKEP-Q---- ---------- --YDAEAPLR FVGDGDVIRL
AfPmt2 ANNDWFIYPN RHEP-E---- ---------- --YDASGPLS FVGDGDVIRL

       ..........560.......570.......580.......590.......600
ScPmt2 VHKSTGRNLH THPVAAPVSK TQWEVSGYGD NVVGDNKDNW VIEIMDQRG-
CaPmt2 MHPQTGRNLH THDIPAPVSK SEYEVACYGN LTIGDPKDNW TVEIMEQAS-
ScPmt3 MHRLTGKNLH THEVPAPISK SEYEVSAYGD VDLGDYKDNW IIEIVEQVG-
ScPmt6 SHKNTGSNLH SHDVPSHVSR GNYEVSGYGS QSVGDEKDDW IVEIVKQMDS
UmPmt2 VHDQTKRNIH SHHVAAPVTK ENLEVSGYGD DQTGDDNDHW VVEVVDDMVH
CnPmt2 VHTQTGRNMH SHAIAAPVTK ESWEVSGYGN LTIGDENDLW IVEVVDDTHT
CaPmt2 NHKNTGSYLR ANAVGAPITS SSYEVSCFGD VESNDWADEW VIEIQSQDQS
SpOgm2 IHPFTNRNLH THKIPAPLNK RMYEVSGYGL GDVGDEKDYW IVNILYDTA-
BcPmt2 VHSQTGRNLH SHDVSAPITK ADKEVSCYGN TTVGDDKDHW TMEVVKDVS-
AnPmtA IHGQTGRNLH SHNIPAPITK NHHEVSCYGN LTIGDDKDHW KVEVVDDVA-
AfPmt2 IHGQTGRNLH SHAIPAPITK SQYEVSCYGN ITIGDEKDHW AVEVVDDVA-

       ..........610.......620.......630.......640.......650
ScPmt2 ------DEDP EKLHTLTTSF RIKNLEMGCY LAQTGNSLPE WGFRQQEVVC
CaPmt2 ------DEDK MRLHPLTSSF RLKNEVMNCY LGVTGTTLPQ WGFRQGEVVC
ScPmt3 ------EEDP TLLHPLSTSF RIKNSILGCY LAQSGKHLPE WGFRQGEVVC
ScPmt6 PNPVYSNENS TILHPVSTFF RLRHKVLGCY LASTGLTYPA WGFKQAEIVC
UmPmt2 G---KVAR-D APVRSLTSRL RLRHKNLGCY MRAANAVLPQ WGWKQVEVSC
CnPmt2 S---KKNNED GRIHSLTTRM RLKHRQLNCY LRAANAVLPQ WGFKQVEVSC
CaPmt2 PDPMFQDEDP SEIHSVSTSF RLKHKQLGCY LATTGKSYPA WGYQQGEVVC
SpOgm2 ------HRDA YNVRSLSTVF QLYNPVVGCY LSSSSSSLPS WGFGQIEMYC
BcPmt2 ------SNDR SKIRTLTTAF RLKHTSLGCY LRAGNVNLPQ WGFKQIEVTC
AnPmtA ------SRDR SRIRTLTTAF RLRHAVLGCY LRAGNTNLPQ WGFKQIETTC
AfPmt2 ------SRDR SRIRTLTTAF RLRHIVLGCY LRAGNVNLPQ WGFKQIETTC

       ..........660.......670.......680.......690.......700
ScPmt2 MKNPFKRDKR TWWNIETHEN ERLPPRPEDF QYPKTNFLKD FIHLNLAMMA
CaPmt2 YKNPFKKDKR TWWNIENNRN AVLPPAPEDF KLPKTKFIRD FIQLNLAMMA
ScPmt3 LKHASKRDKR TWWNIETHEN ERLPQ-GEDF VYPKTSFFRN FMQLNSAMMA
ScPmt6 KDSWSRRDKS TWWNVEDHWN HNLE-TAEDY VPPKSNFWTD FILTNFAMAS
UmPmt2 DKENNPKDQH TWWNIENHWN ERLA-PGDS- QLYKSPFLRD FIHLNVAMMT
CnPmt2 TKENNPKDLH TYWNVESHWN DRLP-AGNA- KLYKSPFWRD FVHLNVAMWT
CaPmt2 KYSVFSRDKN TWWNIEKHVN NKLPLPATEY VPPKPKFWKE FILLNYAMMA
SpOgm2 DPDPDPSNTD TQWNVEEHIN PRLP--EGSI NDYPSSFWSD FLHLNRAMLR
BcPmt2 TKDNNPKDVY THWNVEAHWN EKLP--AADA GAYKSPFIHD FIHLNVAMMT
AnPmtA VKENKPRDVY THWNIETHTN DRLP--PGDP GSYKSPFFKD FVHLNVAMMT
AfPmt2 VKENNPRDVY THWNVESHFN DRLP--PGDP GSYKSPFFKD FIHLNVAMMT

       ..........710.......720.......730.......740.......750
ScPmt2 TNNALVPDPD KFDYLASSAW QWPTLNVGLR LCGWGDDNPK YFLLGTPAST
CaPmt2 TNNALVPDTE KQDDLASSFW QWPTLNVGIR MCGWGPENPK YYMIGSPATT
ScPmt3 TNNALVPNPE KFNGIASSAW QWPTLNVGVR LCEWSEKSIK YFLLGSPASV
ScPmt6 SNNALVPDED KYDSLSSDAW EWPTLHKGLR MCSWAGYITR YYLMGSPFNT
UmPmt2 SNNALIPDAD KEDILASKPF DWPWLWNGLR MNSWDDNSIK FYLLGNPVIW
CnPmt2 SNNALVPDPD KEDILASQPF DWPFLHLGLR MCGWGDHQIK FYLLGTPIIW
CaPmt2 SNNALIPDPD RFDKLSSEWW EWPILNTGLR MNSWGDADIK YFLLGNPLIT
SpOgm2 ANNGLIPDED KLDALRSEAY QWPFLLATLR MCGWGDNQIK YLLVGNPVAY
BcPmt2 SNNALVPDPD KQDDLASYFW QWPILNVGLR MCGWDDNIVK YFLLGNPLVY
AnPmtA SNNALVPDPD KQDDLASKPW QWPILNVGLR MCSWDEKVVK YYLLGNPVVY
AfPmt2 SNNALVPDPD KQDDLASKFW QWPILNVGLR MCSWDDNTIK YYLLGNPFVY

       ..........760.......770.......780.......790.......800
ScPmt2 WASSVAVLAF MATVVILLIR WQRQYVDLRN --PS------ ----NWNVFL
CaPmt2 WTSTVGVILF AFIVLYYLIR WQRQYVDFPS TNPH------ ----KLKLFL
ScPmt3 WPSSIAVCAL IIHVIFLTLK WQRQCVILSD --PV------ ----ERDVFV
ScPmt6 WISTVSLIIF PFIILFILYR WRRQTLYLSD --DQ------ -----IWQIT
UmPmt2 WASSSSLLAF GATWLWYMMR RQRRIHDLSP --AD------ -----WAHFL
CnPmt2 WFSTISLAIG LGLAAWYVAR MQRGYKEWKA --GE------ -----WDHWL
CaPmt2 WISTIALIVC PLYLLVVGIK YQRQWILLSA --TDTSNANP ANSQSLSLLA
SpOgm2 WFATSSLIVF ALFVVGAVLA WRRRVLRWSQ --EA------ -----CDTFH
BcPmt2 WGSTASLGLL GLLVLWYLVR WQRGYDELKQ --SE------ -----IDQIH
AnPmtA WGSTLSLAVF GLLTLWYLVR WQRGYNELSQ --AD------ -----IDHIH
AfPmt2 WGSTFSLGVF GLLIFWYLAR WQRGYKDLNQ --AD------ -----IDHIH
                 VI
       ..........810.......820.......830.......840.......850
ScPmt2 MGGFYPLLAW GLHYMPFVIM SRVTYVHHYL PALYFALIIL AYCFDAGLQK
CaPmt2 MGGIYPMFGW GLHFLPFAIM GRVTYVHHYV PALYFAMLVF CYEVESFSSR
ScPmt3 MAAFYPLLAW LLHYMPFVVM SRVVYAHHYL PTLYFALMIL SYYFDMITKR
ScPmt6 IQGIFPFISW MTHYLPFAMM GRVTYVHHYV PALYFAMLVF GFVLDFTLT-
UmPmt2 YVAKLAAFGW LLHYLPFLVM ARVCYLHHYL PILYFAVLML VHLIDHFVWR
```

```
CnPmt2 WAGKVAFGGW ALHFFPFLIM GRVTYLHHYL PTLYFAVLMA GHILDHFFFA
CaPmt2 ARALLPLAGW VLHYVPFILM GRVKYLHHYV PALYFAIFVA GFIVDAILNL
SpOgm2 YAGIYPFLGW FFNYLPYYIM GRVLYVHHYE PSYALSTFTA AFVVDWFTK-
BcPmt2 YAGLYPVLGW FLHYVPFVAM ARVTYVHHYY PALYFAILVF GFCADWMLR-
AnPmtA YAGLYPVLGW FLHYVPFIAM ARVTYVHHYY PALYYAILTF GFCVDWLTK-
AfPmt2 YSGFYPLLGW ILHYFPFIIM ARVTYVHHYY PALYYAILTF GFCVDWLTQ-
                                                   VII?
       .........860.........870.........880.........890.........900
ScPmt2 WS-------- R---SKCGRI MRFVLYAGFM ALVIGCFWYF SPISFGMEGP
CaPmt2 LN-------- KPNASPVSKL LYLAIYIGLL SLVAGTFWYF RYLSWGMEGP
ScPmt3 WA-------- T---RNTGKF LRLGAYIVYG SIVIAGFFYF SPFSFGMDGP
ScPmt6 ---------- -----RVHWM VKYPIYLSLF GGCIYIYNLF APICQGMHGD
UmPmt2 PSTAVYAFGA SRRKQPLSEA LKNAVFVVSV VAITAAFWWF RGNSYGFKGD
CnPmt2 S--------- ----STRSHT KKLIWFAVWA GVVILSFWWF KDLALGISGN
CaPmt2 DF-------- SYHNNKFQYI FKVVIYSTLY LVICISFWYF KDLSFGMEGS
SpOgm2 ---------- -----KMPKI VRVVVFISLY AIIAGVFIYF KDVTFGMHGP
BcPmt2 ---------- -----NQVKT LQYAIYGVLY ALTIALYIFF MPISWGMEGP
AnPmtA ---------- -----TLNTK VRFLVYGLLY ALVAGVFVYF RVIVFGIEGP
AfPmt2 ---------- -----KMSPV ARGSLYAFLY VIIIGMFVHF RVIVFGIEGP
                                          VII?
       .........910.........920..
ScPmt2 SSNFRYLNWF STWDIADKQE A-
CaPmt2 KEDWKHLKLL ESWRVSDDQY T-
ScPmt3 VDDYAYLAWL PTWQIVEDIR NT
ScPmt6 KAEYLPLQWL STWDIAP--- --
UmPmt2 IKSHKGLKWR KSWNIY---- --
CnPmt2 VNNHWGWGWR SSWNIYN--- --
CaPmt2 SVDYRHLRLL GSWMI----- --
SpOgm2 ASDFHRLRWL NSWNVHD--- --
BcPmt2 NREYSRLKWF DSWRVTD--- --
AnPmtA SQQWRHLNWL SGWRIAN--- --
AfPmt2 SQQWANLNWL SGWRIAN--- --
```

ALIGNMENT OF PMT4 SUBFAMILY MEMBERS

```
      .........10 .........20 .........30 .........40 .........50
ScPmt4 MSVPKKRNHG KLPPSTKDVD DPSLKYTKAA PKCEQVAEHW LLQ-------
SpOgm4 MASKSEKAVK KAQKLSKEPS VELTDTKSSD NVTPKQKSPN STEEDV--SL
UmPmt4 MVDATKAQTL RSRRNDSATA HTTDAVASTR ADHSIVTDVK LGSKSNWGVV
CnPmt4 -----MSLAP RKRRTDRNES PSLPVRSYSD DDKQRTPKPP LSA-------
BcPmt4 MSSQGSVRKR KGGSVPPQAK DASTVAAPHP ELDALVKDIQ AKT-------
AnPmtC MSSSPSLRKR GG------KR EDTPVPSDRS FAPSASQLGA ASR-------
AfPmt4 MSSSPSSSLR KRG-GRKEAY SPLPSDDASS PLSSSKPSVA STN-------
CaPmt4 MSQTLKKRGG NSSGRKSPTT SNIEFDDKKT EFDLNAIVPP K---------

      .........60 .........70 .........80 .........90 .........100
ScPmt4 -PLPEPESRY SFWVTIVTLL AFAARFYKIW YPKEVVFDEV HFGKFASYYL
SpOgm4 NLKTLKAKKF KLAFVLITVL SFITRFWNLN LPGEVVFDEV HFGKFASYYL
UmPmt4 SPHVHSHAAD SLITLVLFAL AAALRLYRIS FPDQVVFDEV HFGKFAAYYL
CnPmt4 PYLRHEYIIS WSTATALTVV ACIVRFWRIA HPDQVVFDEV HFGSFAAQYI
BcPmt4 ----GPEWDY RIALAIITIL AFVTRFWGIS HPNEVVFDEV HFGKFASYYL
AnPmtC ----SSEWDY RLAITILTVL AFITRFYKIS YPDQVVFDEV HFGKFASYYL
AfPmt4 ----QSGWDY RLALVVLTIL AFITRFYKIS YPNEVVFDEV HFGKFASYYL
CaPmt4 ----EPEYKY LAALTLVTLL AIYTRFTKLG TPNKVVFDEV HFGKFASYYL
                    I
      .........110.........120.........130.........140.........150
ScPmt4 ERSYFFDVHP PFAKMMIAFI GWLCGYDGSF KFDEIGYSYE THPAFYIAYR
SpOgm4 QGKYFFDLHP PFAKLLLALV AKLAGYDGHY LFDNIGDNYK DNGVFYVTIR
UmPmt4 RREFHFDVHP PLAKLINAFA GYLAGFDGHF EFDQIGDKYL DNNVFYIRMR
CnPmt4 KREYYFDVHP PLAKMLNGLA AWFVGFDGNF GFDQIGDSYT EASVFYVGMR
BcPmt4 ERTYFFDVHP PFGKLLFALM GWFVGYDGHF HFENIGDSYI DNKVFYVAFR
AnPmtC RRTYFFDVHP PFAKLLLAFT GWLVGYDGHF LFENIGDSYI DNKVFYVALR
AfPmt4 QRTYFFDVHP PFGKLLFAFM GWLIGYDGHF LFDNIGDSYI DNKVFYVALR
CaPmt4 ERTYFFDLHP PFAKLLIAFV GWLIGYDGKF KFEAIGDSYI ENNVFYIAYR

      .........160.........170.........180.........190.........200
ScPmt4 SFNAILGTLT VPIMFNTLKE LNFRAITCAF ASLLVAIDTA HVTETRLILL
SpOgm4 AWPALLSSLV PPVVFLIMKE SGYDLLACIV SSSLVLFDNA HVTEGRLILL
UmPmt4 AVPAIIGSLQ VPLVYAIMRQ SGYAPVIGVF SAALLLFDNA HIAQDRLILL
CnPmt4 SFCAILGTLT IPVVYAIMRE SGYPVGIAAF SAALILFDNG HITQTRLILL
BcPmt4 AMPALLGALT VPTVFAIMWE SGYTLPACVL SASLVLFDNA HIGQTRLILL
AnPmtC AMPAVLGALT IPVVFLIMWE SGYSLPACVL ASGLVLFDNA HVGEDRLILL
AfPmt4 AMPATLGALT IPVVFLIMWE SGYSLPACVL AAGLVLFDNA HVGEDRLILL
CaPmt4 SLSAIQGAAI VPIMFLTMKT LGFSVAACLF SSIIVCFDNA QVTDSRLILL
                 II                           III
      .........210.........220.........230.........240.........250
ScPmt4 DAILIISIAA TMYCYVRFYK CQLRQPFTWS WYIWLHATGL SLSFVISTKY
SpOgm4 DATLLFSMVC AIYCYVRFFK LR-HTPFSRP WWAWLFFTGF FLSCTISTKY
UmPmt4 DAPLILFMML SLYSYIRFYK LR-YNEFSSE WWVWLCATGV NLALTMSCKM
CnPmt4 DAHLVLFMAL SLFCYVKFHQ YR-YQEFSRA WWGWLLATGF WLACTLGCKM
BcPmt4 DATLVLAMAC SLLCYIKFYK LR-HIPFSRK WWKWLLLTGF ALSCDISTKY
AnPmtC DSTLVITMAL SILCYIRFYK LR-HEPFGRK WWKWLLLTGV SLSCVISTKY
AfPmt4 DASLVLSMAL SILCYVRFYK LR-HEPFGRK WWKWLLLTGV SLSCVISTKY
CaPmt4 DATLILSVAL TIFSYSKFST FR-KQPFSSK WWTWLLATGV SLSCVISTKY
                IV
      .........260.........270.........280.........290.........300
ScPmt4 VGVMTYSAIG FAAVVNLWQL LDIKAG---L SLRQFMRHFS KRLNGLVLIP
SpOgm4 VGFFTFLSIG LSVCLELWYL WDIKTG---L TVERFFQHFL ARFFCLIFFP
UmPmt4 VGLFTFITIG SAVAWDLWKL LDIRRG---L EIEHVARHFF ARFFALILLP
CnPmt4 VGLFTFATVG AAVIWDLWEI LDIKKG---H SMSYWTRHFC YRAIGLVIVP
BcPmt4 VGTFAFVTIG SAVAIDLWSL LDINRRQGAL SLPEFGKHFA ARAVALIVVP
AnPmtC VGVFTFVTIG SAVMVDLWNL LDIRRQGGAL TMFEWTKHFA ARFFSLIVVP
AfPmt4 VGVFTFVTIG AAVLVDLWNL LDINRPSGAL SMAHWTKHFA ARAFALIIVP
CaPmt4 VGVFTYLTIG IAVIHELWIL LDYRKG---L TLQEFAKHFF ARLWALIIVP
                                                     V
      .........310.........320.........330.........340.........350
ScPmt4 FVIYLFWFWV HFTVLNTSGP GDAFMSAEFQ ETLKDSPLSV DSKTVNYFDI
SpOgm4 FLFFLFWFYM HFNILTISGP GDSFMSLEFQ ETLSDNPITA NSTILNYYDI
UmPmt4 FGVYLFWFWV HFAILIKSGP GDVFMSSQFQ QTLTGNEMLS NARDIQYFDK
```

```
CnPmt4 FFVYLSFFWV HFKILKFSGP GDSFMSPAFQ ETLEGNELLL NAQEIRYYDT
BcPmt4 FLFYLFWFQV HFAILTKSGP GDDFMSPEFQ ETLSDNAMTA NAVTINYFDN
AnPmtC FFFYLFWFQV HFAILTHSGP GDDFMTPAFQ ETLSDNAMAA QSVSIEYFDT
AfPmt4 FFFYLFWFQV HFAILTRSGP GDDFMTPEFQ ETLSDNLMSA QSIGIQYYDT
CaPmt4 FCIYLYWFYL HFAILTRSGP GDAFMSSEFQ ETLLESPLAA HSKPVQYFDQ

       .........360.......370.........380.........390.........400
ScPmt4 ITIKHQDTDA FLHSHLARYP QRYEDGRISS AGQQVTGYTH P----DFNNQ
SpOgm4 VTIKHMGTNA FLHSHPEKYP IPYDDGRISS GGQQVTGYQF D----DENNY
UmPmt4 LTLMHKNTKA LLHSHVERYP LKYDDGRISS QGQQVTGYPH N----DTNNV
CnPmt4 ISLRHKDTKQ YLHSHEERYP LRYDDGRISS QGQQVTCYPH N----DTNNH
BcPmt4 IQIKHKETHG YLHSHPDTYP LRYDDGRVSS QGQQVTGYPY A----DANNH
AnPmtC ITMRHKDTKV FLHSHSDTYP LRYDDGRISS QGQQVTGYPY N----DTNNH
AfPmt4 ITIRHKDTKV FLHSHWEKYP LRYDDGRISS QGQQVTGYPF N----DTNNH
CaPmt4 ITIKHKDTGA FLHSHQHEYP LRYEDGRISS NGQQVTCVVQ ENAANDPNNQ

       .........410.......420.........430.........440.........450
ScPmt4 WEVLPPH--- -GSDVGKGQA VLLNQHIRLR HVATDTYLLA HDVASPFYPT
SpOgm4 WMILPADHYD PPIEAKLNVP VKNMDYIKLH HVGTNTDLMT HDVASPYHPT
UmPmt4 WQIIPTK--P IPDHDATGRL VHHKDTLRLL HVNTDSYLLT HDVASPLMAT
CnPmt4 WQVIPTK--E -IPESGRGRI VRHNDVIQLK HVNTQTLLLT HDVASPLMPT
BcPmt4 WQVIPAG--T --FEEVAGRP VKNNEVVRLR HVVTNTMLLS HDVASPYYPT
AnPmtC WQIIPTV--P LDETDEKSRK VRNGDIVQLR HVATDTILLT HDVASPYYPT
AfPmt4 WQILPSV--P YPETDRQGHS VKNGDIVQLR HVGTDTILLT HDVASPYYPT
CaPmt4 WEIVPTS--- --EGANKGTK VYTNDIVRFR HVGTGGYLLT HDVASPLKPT

       .........460.......470.........480.........490.........500
ScPmt4 NEEITTVTLE EGDGELYP-E TLFAFQPL-- --KKSDEGHV LKSKTVSFRL
SpOgm4 NEEFTTVSVD ESAGKKHE-Y TLFQVVMS-- --DNTDPQRP LYTKASSFKL
UmPmt4 NEEFTTVPAN DTSRYDDT-L FELHLD---- --EADGTDKV VKSRASWFRL
CnPmt4 NQEFTTVSPD KEDKRNDT-L FKMVL----- --DDAHDGEA WKTLSGHFRL
BcPmt4 NQEFTTVSIE DALGERLN-D TLFELRI--- --ENGKPRQD FKSLQGQFKL
AnPmtC NQEFTTVSHE LADGKRHN-D TLFEIRV--- --EHGKSKQE FRTLSSQFKL
AfPmt4 NQEFTTVSHE LANGERHN-D TLFEIKI--- --ENGKPQQE FRSLSSHFKL
CaPmt4 NEEFTVVYDD VAQQRYNETL FRLRLHVPGS NPKKEKNKKE IKTLATDLRI

       .........510.......520.........530.........540.........550
ScPmt4 FHVDTSVALW THNDELLPDW GFQQQEINGN KKVIDPSNNW VVDEIVNLDE
SpOgm4 IHKLTHVAMW SDPKP-LPDW AFKQLEINGA KNIQTGSIFW TFDDIIGLKD
UmPmt4 VHKNTRVCMW THGEA-LPDW AFNQQEVNGN KNAMDKTCLW YVEDLTPSPE
CnPmt4 IHVPTKVALW THPKA-LPEW AFGQQEVNGN KNAAERSTLW FVNDIVEDGQ
BcPmt4 IHWPSKVAMW THTTP-LPDW AYKQQEINGN KNLAQSSNIW YVDEIPSIPK
AnPmtC VHVPTKVAMW THTTP-LPDW AYKQAEINGN KNVLQSSNIW YVEAIESLEE
AfPmt4 IHVPTRVAMW THTTP-LPEW AFKQAEINGN KNILQTSNLW FVDTIESLEE
CaPmt4 LHVDTVVAMW THNDELLPEW AFNQQEVSGN KKIPDKDNIW NFDLITNLQS

       .........560.......570.........580.........590.........600
ScPmt4 V------RKV YIPKVVKPLP FLKKWIETQK SMFEHNNKLS SEHPFASEPY
SpOgm4 S------RLK KEKKIPKKLP FWKKYLELQL TMFRQNNMLT EFHPYSSNPS
UmPmt4 SPDYEERLKP LAPRKVVKMN FFKKWLELQL QMLQQNAGLT QSHPYATGPI
CnPmt4 GFDFKNRTVH VEPKAVKKRA FIKKWFELQV LMLQHNAGLT STHPYQSTPI
BcPmt4 D----SPRLI KEERKVKSVP FLKKWFELQR AMFYHNNALT SSHPYASEPF
AnPmtC D----SPRLK KEERKVKHLP FWRKYIELQR AMFFHNNALT SSHPYASEPF
AfPmt4 N----SPRAI KKERQVKHLP FWRKYLELQR AMFFHNNALT SSHPYASEPF
CaPmt4 T----DPRNQ YVPKKVKTLP FLRKWWELQM LMFHHNNQLS SEHPFATQPG

       .........610.......620.........630.........640.........650
ScPmt4 SWPGSLSGVS FWTNGDEKKQ IYFIGNIIGW WFQVISLAVF VGIIVADLIT
SpOgm4 DWFTLHHGIA FWAKSEENKQ IYLLGNPIGW WIIAGTVLST TVVAAAEILL
UmPmt4 NWPFLLQGIS FWTQDAGQHQ IYMIGNLVSW WGTILAISIF VGVVGADLLA
CnPmt4 EWPFCLSGIS FWTRNEGQQQ IYMIGNLLAW WICAVSVSVY VGVVAADMLA
BcPmt4 VWPFLLRGVS FWTHNDTRQQ IYFLGNPLGW WLASSLLAVY AGIWAADQLS
AnPmtC QWPFLLRGVS FWTKSDTREQ IYFLGNPVGW WISSSLLAVF AGVIGADQLS
AfPmt4 QWPFLLRGVS FWTKNDTREQ IYFLGNPIGW WIASSLLAVF VGVIGADQLS
CaPmt4 EWPLALSGVS FYNDNTEKKQ IFFIGNIIGF WLEVCFLSIY IGILLADQIT
                                                 VI
       .........660.......670.........680.........690.........700
ScPmt4 RHRGYYALNK ----MTREKL YGPLMFFFVS WCCHYFPFFL MARQKFLHHY
SpOgm4 RQRGIRTLPE ----TVRNHF YRSTMFFYMT YVFHYLPFFI MGRQLFLHHY
UmPmt4 RRRAIYPIPS ----AVRNRL HNSSGFFVMA WACHYLPFFL MSRQLFIHHY
CnPmt4 RRRGIHPIED ----GVRNRL YRNTGFFLGA WAFHYFPFYL MNRQRFLHHY
BcPmt4 LRRGVDALDN ----RTRSRL YNSTGFFFLA WATHYLPFYL MGRQLFLHHY
AnPmtC LRRGVDAVEE IWGPGSRSRL YNSTGFLFLC WAAHYFPFWL MGRQRFLHHY
AfPmt4 LRRGVDALEE IWGRGARSRL YNSTAFLFLC WAAHYFPFWL MGRQRFLHHY
CaPmt4 RRRNVHVLSD ----RARSRL YNTLGFLFVG WAAHYLPFFL MNRQKFLHHY
```

```
       .........710.........720.........730.........740.........750
ScPmt4 LPAHLIACLF SGALWEVIFS DCKSLDLEKD EDI------- --------SGA
SpOgm4 LPAHLAGSLL VGAFIQLACR KSFRSPVSAG VPIPKD---- -----VDEKG
UmPmt4 LPAHVCACLV AGGVLNFVTS ETIQFPISKP GPLLT----- --------PRH
CnPmt4 LPAHLASVLV AGSVMNFILI EAVNYPISIA GPTTR----- --------LR-
BcPmt4 LPAHLASCLI TGALLEFIFN VEPIVDDVPS GVAKKAGAV- -----KKHLP
AnPmtC LPAHVASALV TGALIEFIFN IQPISVPATI PVAADDPTGK GKT--RRFVT
AfPmt4 LPAHVASCMV AGALIEFIFN LQPISVARAV KEDDPTGKSK AHGSAGHFVT
CaPmt4 LPAHLVAALF SGGLVEFICS NNSARP---- ---------- ----------
                VII?
       .........760.........770.........780.........790.........800
ScPmt4 SYERNPKVYV KPYTVFLVCV SCAVAWFFVY FSPLVYGDVS LSPSEVVSRE
SpOgm4 HSKCHRKYGH VIELICTLLL IFVVIYCFTF FAPMTYGDKS LSVDEWTRRK
UmPmt4 YRPRMDNIVP QQARAFMALV IGALIVCFWW LSPLTYGTPG LTNAEVHARR
CnPmt4 --PAVRAKLN KSAKMVIAGL LVVVIGVYLF LSPLTYGQS- MTGEEVNRRK
BcPmt4 AREKLAGQNL MMSWAAVGVI LSIVLFGWYF FLPLTYGYPG LSVPEVNARK
AnPmtC ARERMGVKSI VAGWIASLTI LAATIWGFWF FAPLTYGTPG LDVAQVNARK
AfPmt4 AKERMGRQSL IAGWVATLVI LSLTIWGFCF FAPLTYGTPG LDVAGVNARK
CaPmt4 -NGKPVGVNK YKIIAVVAAC STAIIWFFFY FRPLTYGDVY LTPEEVKARQ
                                VII?
       .........810.........
ScPmt4 WFD-IELNFS K---------
SpOgm4 WLDSWVFQYQ KQN-------
UmPmt4 LLPSWTLHFA K---------
CnPmt4 LLSTWSLHFE AKKTHSMDE
BcPmt4 WLG-YDLHFA K---------
AnPmtC WLG-YDLHFA K---------
AfPmt4 WLG-YDLHFA K---------
CaPmt4 WLD-IKLHYG K---------
```
