# Supplementary material for: Botrytis cinerea Protein O-Mannosyltransferases Play Critical Roles in Morphogenesis, Growth, and Virulence
Source: PLoS One. 2013 Jun 6;8(6):e65924. doi: 10.1371/journal.pone.0065924 (PMC3675079; doi:10.1371/journal.pone.0065924)
Supplement: Table S1 — Protein sequences used in this work. Protein sequences used in the comparative studies displayed in Figures 1 and S2. (PDF) [file pone.0065924.s005.pdf]

# **Table SI.** Protein sequences used in this study

| Organism | GenBank acc. number | Gene name | PMT subfamily |
|---|---|---|---|
| *Saccharomyces cerevisiae* | NP_010188 | *ScPmt1* | 1 |
| | NP_009379 | *ScPmt2* | 2 |
| | NP_014966 | *ScPmt3* | 2 |
| | NP_012677 | *ScPmt4* | 4 |
| | NP_010190 | *ScPmt5* | 1 |
| | NP_011715 | *ScPmt6* | 2 |
| *Schizosaccharomyces pombe* | NP_593237 | *SpOgm1* | 1 |
| | NP_594135 | *SpOgm2* | 2 |
| | NP_596807 | *SpOgm4* | 4 |
| *Ustilago maydis* | XP_762320 | *UmPmt1* | 1 |
| | XP_761621 | *UmPmt2* | 2 |
| | XP_761580 | *UmPmt4* | 4 |
| *Botrytis cinerea* (B05.10) | XP_001548518 | *bcpmt1* | 1 |
| | XP_001558317 | *bcpmt2* | 2 |
| | XP_001558914 | *bcpmt4* | 4 |
| *Cryptococcus neoformans* var. neoformans | XP_570521 | *CnPmt1* | 1 |
| | XP_567365 | *CnPmt2* | 2 |
| | XP_570292 | *CnPmt4* | 4 |
| *Aspergillus nidulans* | XP_662365 | *AnPmtB* | 1 |
| | XP_662709 | *AnPmtA* | 2 |
| | XP_659063 | *AnPmtC* | 4 |
| *Aspergillus fumigatus* | XP_754961 | *AfPmt1* | 1 |
| | XP_754961 | *AfPmt2* | 2 |
| | XP_747257 | *AfPmt4* | 4 |
| *Candida albicans* | XP_716993 | *CaPmt1* | 1 |
| | XP_719907 | *CaPmt2* | 2 |
| | XP_714280 | *CaPmt4* | 4 |
| | XP_719311 | *CaPmt5* | 1 |
| | XP_717283 | *CaPmt6* | 2 |
