# Supplementary material for: Botrytis cinerea Protein O-Mannosyltransferases Play Critical Roles in Morphogenesis, Growth, and Virulence
Source: PLoS One. 2013 Jun 6;8(6):e65924. doi: 10.1371/journal.pone.0065924 (PMC3675079; doi:10.1371/journal.pone.0065924)
Supplement: Table S2 — Oligonucleotides used in this study. (PDF) [file pone.0065924.s006.pdf]

Table SII. Oligonucleotides used in this study

Oligonucleotide	Sequence (5’ – 3’)
Nour-Hyg-FOR[a]	CTCTAGAGCCGCATTCCC
Nour-Hyg-REV[a]	TAAGCTTGATATCTGTTAGTAATC
Pmt1-5’-For[a]	CCACAGCAACTCGTTCGC
Pmt1-5’-Rev[a]	GCCCGAATCGGGAATGCGGCTCTAGAGTACTTCAGATGAAAATCG
Pmt1-3’-For[a]	GATGATTACTAACAGATATCAAGCTTATCACAGGAGACAGTACGG
Pmt1-3’-Rev[a]	AGCCAGCCATAACTTGGC
Pmt1-Nest- FOR[b]	AAGATTGTTACAGCAAGC
Pmt1-Nest-REV[b]	CGTTTGCTATGATACTCG
Pmt2-5’-FOR [a,c]	AAAGAGTGGCAATTTGGC
Pmt2-5’-REV[a,c]	GCCCGAATCGGGAATGCGGCTCTAGAGCTCGAATGTCTGCGAGG
Pmt2-3’-FOR[a]	GATGATTACTAACAGATATCAAGCTTACTTAACCACTGCTTTCCG
Pmt2-3’-REV[a]	AACTGGGTACAGTCCAGC
Pmt2-Nest-FOR[b]	AAGTAGACTACTATGCAAGG
Pmt2-Nest-REV[b]	TTAAGCTCGTCATACCCG
Pmt4-5’-FOR[a]	GGCTAATTTTAGCCTCCCGCCCGAATCGGGAATGCGGCTCTAGAGAAGAAAT CCTAAGGGACG
Pmt4-3’-FOR[a]	GATGATTACTAACAGATATCAAGCTTATCGAAGTAGTATTCAAGG
Pmt4-3’-REV[a]	TGTCTTGCTAAACCCACG
Pmt4-Nest-FOR[b]	AATGTGAATGTTGATCTCG
Pmt4-Nest-REV[b]	AGTCTTTTCTAACCTTCC
Hyg-RW[c]	GAGTTCAGCCATGGATCG
pmt1-FW[c]	GGAAGAGGTTATTTGCTGC
pmt1-RV[c]	CATCGAATACCACACTGC
pmt2-FW[c]	GCTCGTAGGAGAATTGACG
pmt2-RV[c]	CGCACCAGAAGCAACAGC
pmt4-FW[c]	CGGAGCAGTTGTCAGAGC
pmt4-RV[c]	AACTGAACCACCTTTCCG
Pmt1_RT_FW[d]	AAAACTGTCACACCGCGC
Pmt1_RT_RV[d]	TCCATCACCGTCTCCTGG
Pmt2_RT_FW[d]	GGAACGAGAAATTGCCTGC
Pmt2_RT_RV[d]	GCACAAGTGCGTTGTTCG
Pmt4_RT_FW[d]	GATAACCGTACCCGATCCC
Pmt4_RT_RV2[d]	CAAGAAAAGTTGACGGCCC

[a]Oligonucleotides used to amplify individual fragments to be fused by PCR to generate the knocked-out *bcpmt* genes. Underlined nucleotides indicate the regions of the *bcpmt* amplicons homologous to the hygromycin resistance cassette, which were necessary for the fusion PCR.
[b]Oligonucleotides used to amplify each final gene replacement cassette used to transform *B. cinerea.*
[c]Oligonucleotides used to check Δ*bcpmt* mutant strains.
[d]Oligonucleotides used in Q-RT-PCR.
